# Bio‐Inspired Bidirectional Winding Origami With Programmable Modular Reconfigurability and Scalability

**DOI:** 10.1002/advs.76652

**Published:** 2026-07-20

**Authors:** Wenyao Zhang, Chunlong Wang, Mingli Liu, Chuang Shi, Hongwei Guo, Rongqiang Liu

**Affiliations:** ^1^ School of Mechatronics Engineering Harbin Institute of Technology Harbin China; ^2^ National Key Laboratory of Aerospace Mechanism Harbin China

**Keywords:** bidirectional winding, bio‐inspired design, deployable structures, modular scalability, motion programmability, origami

## Abstract

Origami‐enabled winding deployable structures offer a promising route to ultralight, compact, and stiff large‐scale spacecraft systems. Existing winding origami, however, provides only limited discrete winding‐angle options and suffers from strong scale–angle coupling, forcing trade‐offs between structural size and functional programmability. Here, we present a bio‐inspired origami architecture with bidirectional winding, modular reconfiguration, and scalable extension. Inspired by earwig hindwings, a double axial‐folding origami unit is designed and assembled into a circumferential array to form a bidirectionally winding origami foundation. Hybridization with Flasher origami further produces two coupled origami modes that preserve bidirectional winding while inheriting modular scalability. Building on this concept, a bidirectional‐winding multilayer origami structure with programmable winding kinematics is developed, where a discrete set of attainable winding angles is generated by assigning layer numbers without changing the central hub or outer boundary size. The resulting design space forms a rich library of winding‐angle candidates for programmable deployment. A membrane deployment mechanism is then demonstrated, experimentally confirming bidirectional winding and markedly reduced rotation demand. This work establishes a programmable and scalable platform for large‐area forced‐deployment systems in civil and aerospace engineering.

## Graphical abstract

A bio‐inspired bidirectional‐winding origami platform enables scalable and programmable deployable structures. Double axial folding inspired by earwig hindwings and Flasher‐based hybridization create modular multilayer architectures with diverse attainable winding angles under fixed boundary conditions. The demonstrated membrane deployment mechanism reduces rotation demand, highlighting a promising route toward large‐area forced‐deployment systems.

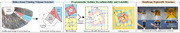

## 1 Introduction

Origami‐enabled deployable space structures combine low mass [1], high packing efficiency [2], and high stiffness [3], making them promising structural platforms for large spacecraft and complex payload systems [4]. Large‐scale space missions impose stringent fairing‐volume constraints, requiring compact stowage, reliable deployment, and controllable motion paths. Research has therefore shifted toward origami architectures that can be compactly stowed and reliably deployed into stable configurations [5]. Conventional non‐winding patterns, such as Miura–ori [6], primarily rely on in‐plane expansion and layered compaction [7], which may constrain packing efficiency and ordered deployment in certain large‐area deployment scenarios. Winding origami offers a complementary route by enabling compact stowage and ordered deployment through continuous spiral motion, thereby providing a potential route toward high deployment‐to‐stowage ratios and controllable deployment paths [8].

Geometry design and foldability remain central challenges in winding origami. Palmer and Shafer [9] introduced the Flasher pattern through asymmetric twist folding, establishing a rotationally symmetric prototype with radial collapse and rapid deployment capabilities. Nojima [10] subsequently proposed an analytical framework for wrapping origami inspired by biological helices, enabling high packing ratios. Lang and co‐workers [9] further developed unidirectional winding‐folding strategies, providing theoretical support for complex winding configurations. Recent studies have expanded this design space: Wong [11] achieved textured, figurative, and polyhedral winding structures by tuning layer‐thickness distributions; Zhang et al. [12] established mathematical models for planar multi‐unit wrapping origami and improved global foldability by optimizing unit geometry and eliminating boundary‐crease mismatch; and Wang et al. [13] proposed Bloom origami assembled from wedge‐shaped units with explicit design and foldability criteria. These studies establish a solid foundation for Flasher‐based and rotationally symmetric winding origami. However, most existing architectures still follow unidirectional global rotation or a single dominant winding path, whereas coordinated bidirectional winding within one deployable architecture remains less explored.

Bioinspired origami offers a complementary route for developing highly compact deployable structures. The earwig hind wing, with its remarkable area expansion ratio, coordinated crease network, and rapid reversible folding, has become a representative biological model for deployable structures [14, 15]. Faber et al. [16] revealed how wing‐vein geometry, folding topology, and elastic energy storage–release mechanisms jointly enable compact stowage and rapid large‐area deployment, leading to spring‐origami concepts for biomimetic mechanisms and space deployable structures. Saito et al. [17] combined flat‐foldability constraints with X‐ray micro‐CT‐derived wing geometries to develop reproducible geometric design tools for complex fan‐shaped crease patterns. Ishiguro et al. [18] integrated earwig‐inspired fan folding with compliant origami hinges to fabricate lightweight foldable wings for micro air vehicles, and wind‐tunnel tests verified their high folding ratio and lift performance. Existing earwig‐inspired structures mainly emphasize geometric reconstruction [16, 17], deployment‐path analysis [14, 19], or individual biomimetic folding units [18, 20]. Their reverse‐rotation potential, local unwinding behavior, and multi‐region cooperative winding mechanisms have not yet been fully translated into programmable winding‐origami architectures. The earwig hind wing therefore provides not only a deployable geometric prototype but also a biokinematic basis for developing origami units capable of bidirectional winding.

Understanding the deployment kinematics of complex origami systems requires systematic modeling of their motion paths, mobility, and singular configurations. Chen et al. [21] established a systematic model for Flasher units, quantified deviations between ideal and physical geometries, and revealed energy‐landscape‐driven configuration evolution. Cai et al. [22] proposed a Jacobian‐ and higher‐order‐expansion‐based method to identify degrees of freedom and singularities in rigid origami, uncovering multiple wrapping modes under different mountain–valley assignments. Feng et al. [23] analyzed the rigid foldability of square twist patterns, derived kinematic equations, and enhanced structural stiffness by adding creases. Xi et al. [24] simulated a scalable winding origami structure and identified sequential layer‐by‐layer folding under single‐degree‐of‐freedom actuation. Meloni et al. [25] developed a Kangaroo‐based computational framework that combines incremental dihedral‐angle updates with dynamic relaxation for high‐fidelity folding‐path prediction. These approaches effectively support mobility identification, singularity analysis, trajectory prediction, reachability assessment, and design optimization. Nevertheless, existing studies mainly focus on the motion paths of prescribed crease patterns, leaving systematic kinematic strategies for constructing origami systems with programmable bidirectional winding behavior largely underexplored.

A growing body of outstanding research has advanced origami structures from configuration design and mechanism‐principle exploration toward engineering validation. Representative demonstrations include ultra‐compact and thermally stable starshade central rings and petal‐like shields based on wrapping origami [26], membrane reflector packaging that combines rolling, folding, and sliding‐assisted creases to reduce plastic‐crease‐induced surface errors [27], Flasher‐based deployable antennas enabled by kirigami constraint release, prototype testing, and electromagnetic tuning [28, 29], and lightweight composite rocket airbrakes based on wrapping origami that remain safe under aerodynamic loads [30]. These studies confirm the engineering potential of winding origami for high‐packing‐ratio space structures. Yet large‐scale deployment requires not only compact stowage, but also low‐disturbance motion, simple actuation, and repeatable reliability. Current wrapping and winding origami designs are dominated by unidirectional winding and global rotational deployment [31, 32]. Scaling such mechanisms may increase angular‐momentum demand, torsional coupling, and control difficulty, while manufacturing errors can induce trajectory drift, jamming, or locking [33, 34]. Strong coupling between overall size and winding angle [35] further increases actuation stroke, internal strain, and stability requirements [4]. Large winding angles may also intensify self‐contact, local crease locking [36, 37], energy dissipation, and deployment resistance [31, 38]. Architectures capable of redistributing or partially counteracting local rotations through bidirectional winding are therefore desirable for improving deployment reliability and motion controllability.

Here, we present a bio‐inspired bidirectional‐winding multilayer origami architecture that enables highly programmable winding kinematics, modular reconfiguration, and scalable extension. Drawing on the earwig hindwing's second axial crease, we map its crease features into a parametric polyline network and construct a bidirectional‐winding origami with two‐stage axial folding. This two‐stage folding/unfolding sequence induces counter‐rotating winding of the inner and outer regions, thereby embedding intrinsic bidirectionality. Integrating the design with Flasher origami yields two coupled hybrid modes that preserve bidirectional winding while leveraging the flasher's scale‐expansion capability and enhanced motion performance. A multilayer FO–BW–FO hybrid framework further enables programmable winding‐angle configurations, in which a discrete set of attainable winding angles can be generated by assigning layer numbers without changing the central hub or outer boundary size. Within this discrete design space, a rich library of winding‐angle candidates is formed, providing multiple options for programmable deployment design. We further translate this concept into an engineered membrane deployment system by developing a scissor‐mechanism‐based controllable drive and synchronized deployment device. Experiments confirm bidirectional winding during deployment and demonstrate a substantial reduction in the required rotation. This work establishes a programmable, scalable platform for large‐area forced‐deployment systems, with broad potential for space deployable mechanisms and architectural deployable structures.

## 2 Results and Discussion

### 2.1 Geometric Design of the Double Axial‐Folding Origami Unit

**FIGURE 1:**
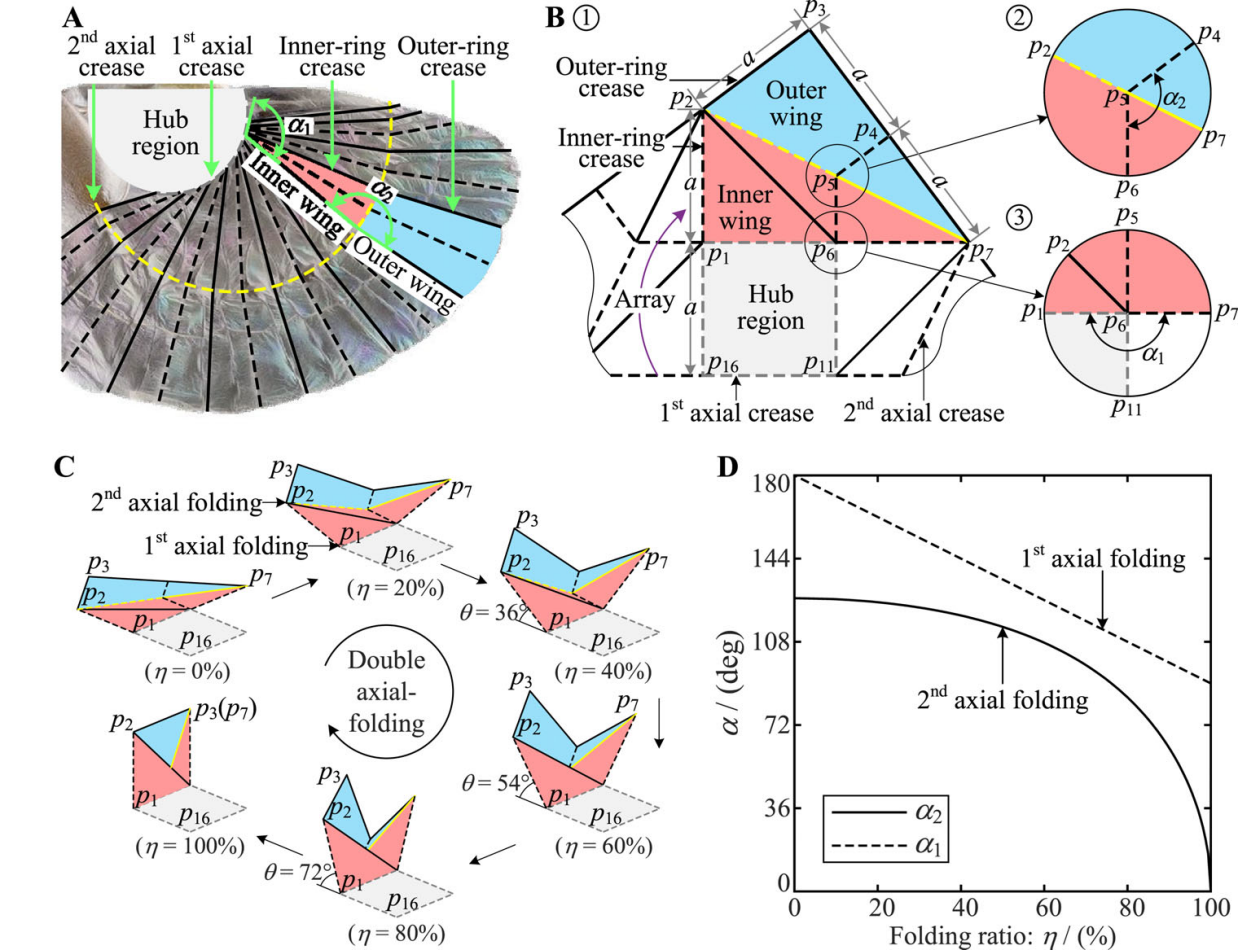
Earwig hindwing–inspired origami unit with double axial folding. (A) Double axial‐folding features of the earwig hindwing. (B) Crease pattern and geometric parameters of the double axial‐folding origami unit. (C) Stage‐wise morphological evolution during double axial folding. (D) Angle–evolution curves characterizing double axial folding.

The earwig hindwing exhibits an exceptionally high folding ratio and a highly intricate geometry [16]. It combines efficient stowage with robust, repeatable deployment [17], enabled by a crease network that constrains the DOFs and enforces a well‐defined folding–unfolding pathway. Motivated by this capability, we analyze its crease pattern to extract transferable geometric rules. As shown in Figure 1A, the hindwing consists of a hub, an inner wing, and an outer wing, connected by two axial creases and supplemented by multiple circumferential creases across the inner and outer wings. This architecture coordinates one fanwise fold followed by two axial creases, yielding an extremely compact stowed state while preserving an ordered, reversible deployment trajectory.

Inspired by the hindwing's architecture, we abstract its crease features into a controllable network and develop a double axial‐folding origami unit (Figure 1B①). First, the relatively rigid central region of the hindwing is mapped onto a square domain $p_{1}p_{6}p_{11}p_{16}$ with side length a; its boundary serves as the primary axial crease and the main folding backbone. Next, the outer and inner wing regions are represented by two right isosceles triangles with leg lengths a and 2a, respectively. Following the hindwing crease topology, we define the second axial creases $p_{2}p_{5}$ and $p_{5}p_{7}$, the outer circumferential creases $p_{2}p_{3}$ and $p_{4}p_{5}$, and the inner circumferential creases $p_{1}p_{2}$, $p_{5}p_{6}$, and $p_{6}p_{7}$. Hub‐centered rotational tiling and fold compatibility are ensured by adding an auxiliary crease $p_{2}p_{6}$. The double axial‐folding origami unit can be interpreted as a serial combination of a single‐vertex four‐crease module (Figure 1B②) and a single‐vertex five‐crease module (Figure 1B ③). Synchronized hub‐centered tiling requires creases $p_{1}p_{2}$ and $p_{6}p_{7}$ to obey a rotational‐symmetry constraint, which reduces the kinematic degrees of freedom from 2 to 1.

The double axial‐folding behavior of this origami unit can be quantified by defining θ as the acute angle between $p_{1}p_{2}$ and $p_{1}p_{16}$, and the folding ratio as $\eta =\theta /90^{\circ }\times 100\%$. The folding trajectory is then divided into η‐based stages, enabling stage‐resolved analysis of geometric evolution (Figure 1C). The node‐coordinate calculation is provided in Supporting Information S6. The first axial crease mainly lifts the unit out of the plane, whereas the second axial crease compacts the pattern from a flat to a densely folded state. In the initial stage (η=0%–40%), the unit elevates rapidly under the first axial crease and then slows, while the second axial crease steadily increases compaction: the inner and outer wings compress toward the hub footprint, converging into a square region comparable to the projected hub area, with a monotonically increasing compaction rate. This staged geometric evolution is further characterized using two angular metrics associated with the two axial creases.

Specifically, $\alpha_{1}$ is defined as $∠p_{1}p_{6}p_{7}$ (Figure 1B③) and $\alpha_{2}$ as $∠p_{4}p_{3}p_{6}$ (f1B②), and their evolution is plotted in Figure 1D. Under the rotational‐symmetry constraint, that is, synchronized folding, creases $p_{6}p_{7}$ and $p_{1}p_{2}$ rotate in lockstep; $\alpha_{1}$ therefore decreases linearly with η, from $180^{\circ }$ to $90^{\circ }$. The out‐of‐plane height follows geometric projection and is captured by $\cos\alpha_{1}$, so a linear $\alpha_{1}\left(\eta \right)$ yields a decreasing height‐growth rate with increasing η, consistent with the observed slowdown in elevation. In contrast, $\alpha_{2}$ decreases nonlinearly and accelerates with η, exhibiting a near‐exponential trend and a sharper change near the end of folding. This behavior confirms the progressively accelerating compaction governed by the second axial crease. As $\alpha_{2}\to 0^{\circ }$, the unit approaches near‐complete overlap, reaching an extremely compact limit state.

### 2.2 Bio‐inspired Bidirectional Winding Origami Structure

**FIGURE 2:**
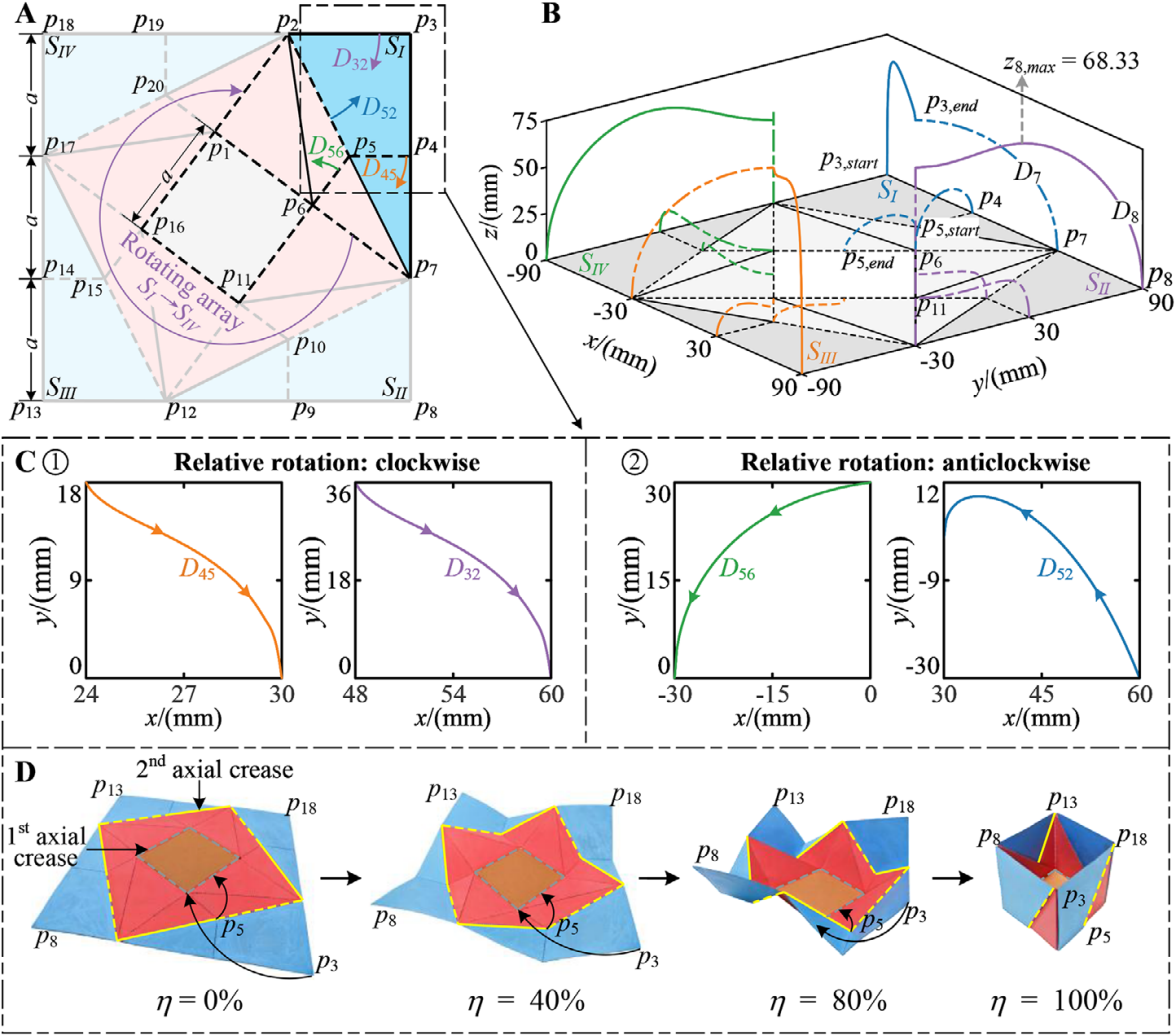
Bidirectional winding origami structure generated from the double axial‐folding unit. (A) Generation method and resulting crease pattern of the bidirectional winding origami structure. (B) Nodal trajectories of the origami structure from the deployed planar state to the fully compact state; the left–right order of $p_{3}$ and $p_{5}$ inverts, indicating counter‐winding during folding. (C) Planar (xy) projections of relative trajectories between selected nodes; clockwise winding of the outer wing (e.g., $D_{45}$, $D_{32}$) and anticlockwise winding of the inner wing (e.g., $D_{56}$, $D_{52}$) together confirm bidirectional winding. (D) Folding process of a paperboard prototype (at representative folding ratios 0%, 40%, 80%, and 100%), validating bidirectional winding between the inner and outer wing regions.

The double axial‐folding origami unit is defined as sector $S_{I}$ and replicated by three successive $90^{\circ }$ rotations about the central hub, generating sectors $S_{II}$, $S_{III}$, and $S_{IV}$. This procedure yields a crease pattern with an overall side length of 3a, termed the bidirectional winding origami structure (Figure 2A). The folding kinematics from the fully deployed planar state to the fully compact state are captured by the trajectories of all nodes (Figure 2B), where a=60mm and $D_{i}$ denotes the absolute displacement trajectory of node $p_{i}$. The related formulas for calculating the node coordinates are provided in Supporting Information S6. Trajectory $D_{7}$ forms a $90^{\circ }$ circular arc, indicating that crease $p_{6}p_{7}$ undergoes fixed‐axis rotation about crease $p_{6}p_{11}$. The first half of $D_{8}$ is approximately circular and reaches the maximum height $z_{8,\max}=68.334\;\mathrm{mm}$, corresponding to the largest z‐direction lift relative to the hub during folding. Subsequently, $D_{8}$ exhibits an approximately S‐shaped descent, indicating that the rate of z‐decrease first increases and then decreases. The outer boundary nodes also undergo a clear relative reordering: $p_{3,\mathrm{start}}$ lies to the left of $p_{5,\mathrm{start}}$, whereas $p_{3,\mathrm{end}}$ shifts to the right of $p_{5,\mathrm{end}}$. This left–right reversal indicates opposing lateral‐displacement trends during folding.

Geometric constraints on the outer‐contour node $p_{3}$ govern this inversion, as its motion is coupled to $p_{2}$, $p_{4}$, $p_{5}$, and $p_{6}$. Absolute trajectories alone do not resolve the swapping mechanism between $p_{3}$ and $p_{5}$; we therefore examine inter‐nodal relative motion to identify its geometric origin. The folding motion is organized around the central square hub, so the global state is dominated by in‐plane (xy) kinematics. Accordingly, in‐plane relative trajectories among $p_{2}$–$p_{6}$ are extracted (Figure 2C), where $D_{ij}$ denotes the trajectory of $p_{i}$ relative to $p_{j}$ and arrows indicate motion direction. In Figure 2C ①, $D_{45}$ and $D_{32}$ trace clockwise paths in the planar projection; their S‐shaped profiles indicate substantial curvature and progressive reorientation of $p_{4}$ relative to $p_{5}$ and $p_{3}$ relative to $p_{2}$. As $p_{4}$ and $p_{3}$ lie on the outer wing, these trajectories indicate clockwise winding of the outer wing. In Figure 2C ②, $D_{56}$ follows an anticlockwise arc, indicating that $p_{5}$ rotates anticlockwise relative to $p_{6}$ with a smaller in‐plane displacement. Trajectory $D_{52}$ exhibits a pronounced late‐stage bend, indicating a direction reversal in the in‐plane motion of $p_{5}$ relative to $p_{2}$. Although $p_{5}$ lies on the second axial crease, its relative motion opposes that of the outer wing, indicating anticlockwise winding of the inner wing. Together, these trajectories confirm bidirectional winding, with the inner and outer wings winding in opposite directions during folding.

This bidirectional winding behavior is further validated experimentally using a 0.2mm‐thick paperboard prototype of the same size (Figure 2D). At a folding ratio of 0%, the structure is fully deployed and planar. As the folding ratio increases from 0% to 40%, out‐of‐plane undulations appear and a three‐dimensional folded shape forms; node $p_{5}$ exhibits a slight anticlockwise rotation, whereas node $p_{8}$ exhibits a slight clockwise rotation. At η=80%, the structure contracts substantially, facets move into proximity, and the rotations of $p_{5}$ and $p_{8}$ intensify. At full closure (100%), the relative positions of $p_{5}$ and $p_{8}$ invert compared with the initial state, confirming bidirectional winding of the inner and outer wing regions during folding.

### 2.3 Programmable Modular Expansion of a Hybrid Bidirectional Winding Origami Structure

#### 2.3.1 Modular Expansion Design Via Hybridized Flasher Origami

Flasher origami shares key commonalities with bidirectional winding origami in crease‐length scaling and structural organization, and it additionally supports outward modular expansion. Leveraging these shared features, we develop a modular expansion strategy that integrates the bidirectional winding behavior of bidirectional winding origami with the scalability of Flasher origami.

**FIGURE 3:**
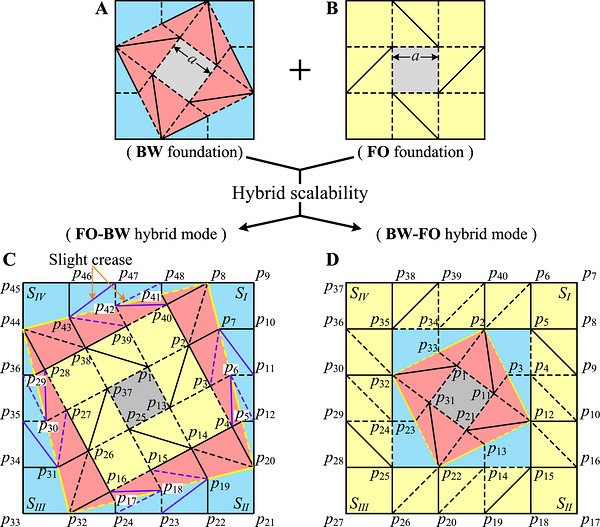
Programmable modular hybridization of (A) bidirectional winding origami (denoted as the BW foundation) and (B) Flasher origami (denoted as the FO foundation). Resulting hybrid origami structures: (C) FO–BW hybrid mode (FO outside BW), and (D) BW–FO hybrid mode (BW outside FO).

As shown in Figure 3, we consider a bidirectional winding origami structure and a conventional Flasher origami structure with the same hub side length a, denoted as the BW prototype (Figure 3A) and FO prototype (Figure 3B), respectively. An “outer‐layer–inner‐layer” convention is adopted to specify the hybrid layouts. When the BW prototype constitutes the outer layer and the FO prototype constitutes the inner layer, the configuration is termed the FO–BW hybrid mode (Figure 3C). Fold compatibility across the BW–FO interface is achieved by adding minor transition creases that preserve geometric coordination throughout folding. Conversely, when the FO prototype forms the outer layer and the BW prototype forms the inner layer, the configuration is termed the BW–FO hybrid mode (Figure 3D). A unified node‐labeling scheme is used for both hybrid configurations in the following analyzes.

#### 2.3.2 Folding‐Induced Bidirectional Winding Characteristics of the FO–BW Hybrid Mode

The FO–BW hybrid mode demonstrates the feasibility of the proposed modular hybridization strategy, and its bidirectional winding behavior is characterized experimentally. A 0.2‐mm‐thick FO–BW hybrid paperboard sample is fabricated, featuring a central hub with side length a=60mm and an overall footprint of 180mm×180mm. The same folding ratio η is used to quantify folding progress, and θ is defined as the acute dihedral angle between creases $p_{1}p_{2}$ and $p_{1}p_{37}$.

**FIGURE 4:**
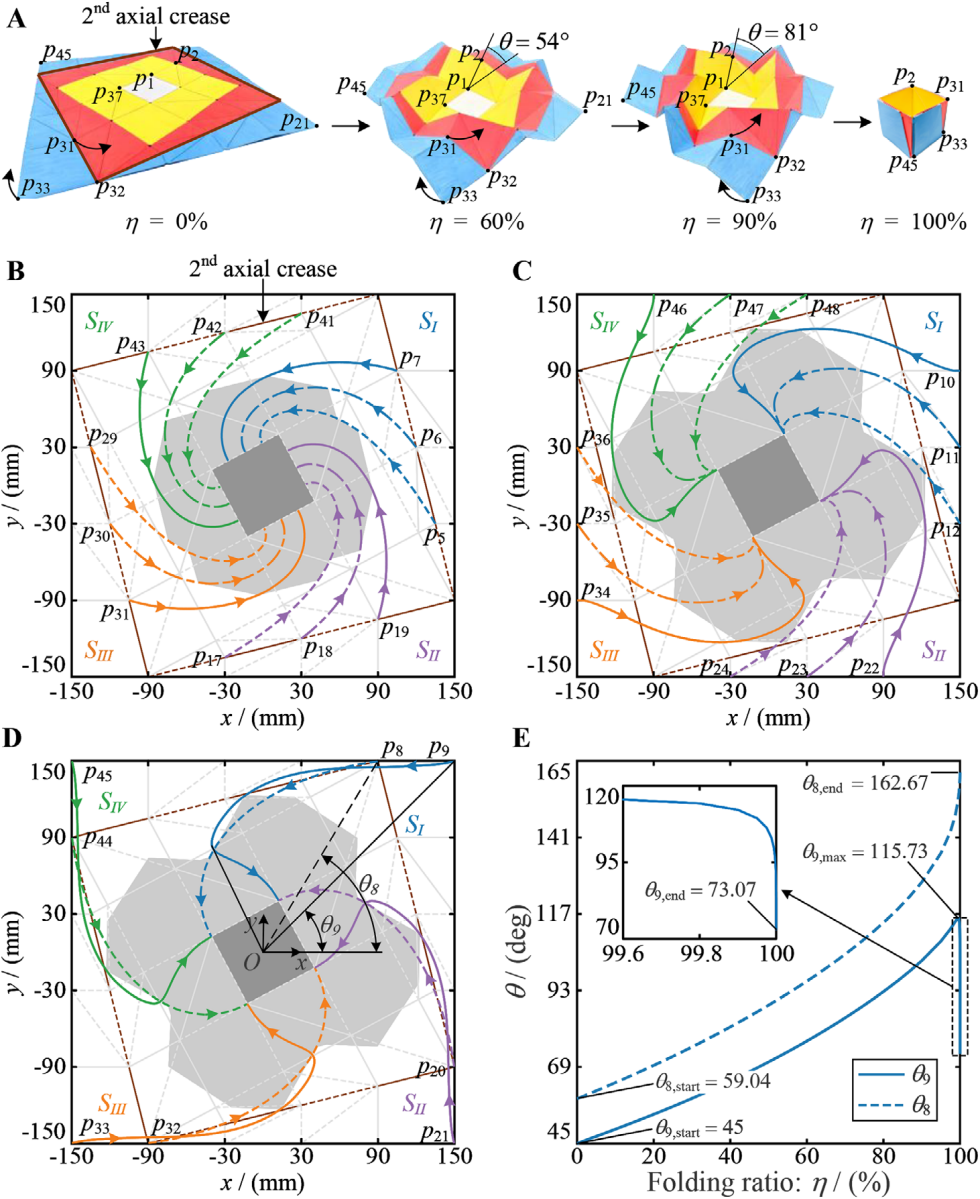
Folding‐induced bidirectional winding characteristics of the FO–BW hybrid origami structure. (A) Folding process of a paperboard prototype (at representative configurations η=0%, 60%, 90%, and 100%). (B) Projection of the trajectory of the 2^nd^ axial‐crease node in the outer BW region onto the xy plane, exhibiting an anticlockwise, inward‐spiraling motion. (C) Projection of the trajectory of the outer‐boundary nodes, showing that nodes adjacent to the second axial crease maintain an anticlockwise inward spiral, whereas more distant nodes reverse their angular direction in the terminal stage (η>90%). (D) Comparison of representative projected corner trajectories, highlighting the second axial crease as a kinematic demarcation. In (B)–(D), the light‐ and dark‐gray shaded regions denote the projected areas in the xy plane enclosed by the selected nodes at η=90% and 100%, respectively. (E) Angular metrics $\theta_{8}$ and $\theta_{9}$ for corners $p_{8}$ and $p_{9}$ as functions of η, showing that the FO–BW hybrid mode retains the BW‐region bidirectional winding signature and enables partial self‐unwinding.

Figure 4A shows the configurations as η increases from 0% to 60%, 90%, and 100%. The inner FO region evolves smoothly, whereas the outer BW region transitions from gradual to accelerated closure, most evident at η=90%–100%, where the envelope volume drops rapidly. The underlying closure mechanism in this terminal stage was assessed by examining nodal rotations. At η=60%, $p_{31}$ rotates anticlockwise, and $p_{33}$ rotates anticlockwise overall with a slight local counter‐rotation relative to crease $p_{31}p_{32}$. At η=90%, the rotational contrast becomes more pronounced. As η increases from 90% to 100%, most of the remaining rotation occurs concurrently with the sharp volume contraction, confirming this interval as the final rapid‐collapse phase. Geometric coupling with the FO region further enhances the late‐stage anticlockwise rotation of the BW node on the second axial crease (represented by $p_{31}$).

A theoretical model is introduced to reveal the geometric origin of this amplification effect. The FO–BW hybrid mode nominally possesses two degrees of freedom, represented by the incremental x‐displacements of nodes $p_{2}$ and $p_{8}$, denoted as $dx_{2}$ and $dx_{8}$. The paperboard folding process exhibits strong kinematic coupling between these coordinates: variations in $dx_{2}$ are accompanied by synchronous changes in $dx_{8}$. Accordingly, $dx_{8}=3dx_{2}$ is adopted, allowing the folding path to be parameterized by a single variable.

Figure 4B–D shows the projected trajectories of the selected nodes on the xy plane in the FO–BW hybrid mode. The related formulas for calculating the node coordinates are provided in Supporting Information S7. The variation of these nodes from η=90% to 100% is highlighted by rendering the xy‐plane projections of the origami region enclosed by the selected nodes at the two states in different colors: the light‐gray region corresponds to the projection at η=90%, whereas the dark‐gray region corresponds to that at η=100%. Both projections are obtained on the xy plane along the viewing direction defined by the elevation angle $\beta =90^{\circ }$. The detailed construction of the light‐ and dark‐gray regions is provided in Figure S1. The markedly smaller area of the dark‐gray region confirms the rapid terminal contraction occurring as η increases from 90% to 100%.

Figure 4B shows the node trajectories along the second axial crease in the outer BW region. The nodes move along curved inward trajectories toward the central hub, exhibiting a pronounced anticlockwise inward‐spiraling contraction. Located along the outward continuation of the inner FO region, these nodes inherit the FO‐region winding mechanism. Their larger initial radial distance yields greater angular accumulation, making the rotation more evident; this also accounts for the pronounced anticlockwise rotation of node $p_{31}$ in Figure 4A at later stages.

Figure 4C shows the trajectories of selected nodes on the outer boundary. Nodes located near the second axial crease (e.g., $p_{12}$, $p_{24}$, $p_{36}$, and $p_{48}$) follow a regular anticlockwise inward spiral throughout folding. By contrast, nodes farther from this axial crease (e.g., $p_{10}$, $p_{22}$, $p_{34}$, and $p_{46}$) follow an initial spiral, but at η>90% exhibit a pronounced tangential reversal, switching from anticlockwise to clockwise and producing a terminal turnback. This behavior indicates that the second axial crease acts as a geometric demarcation that truncates the anticlockwise spiral contraction inherited from the inner FO region, preventing its outward continuation. In addition, the symmetry constraint imposed by the second axial crease drives late‐stage clockwise deflection of the outer‐boundary nodes; this reversed tendency becomes more pronounced with increasing distance from the second axial crease.

Figure 4D compares the trajectories of two representative corner types: corners on the second axial crease ($p_{8}$, $p_{20}$, $p_{32}$, and $p_{44}$) and corners on the outer boundary ($p_{9}$, $p_{21}$, $p_{33}$, and $p_{45}$). The trajectories of $p_{8}$, $p_{20}$, $p_{32}$, and $p_{44}$ remain smooth and directionally consistent, maintaining a continuous anticlockwise inward contraction throughout folding. In contrast, $p_{9}$, $p_{21}$, $p_{33}$, and $p_{45}$ show pronounced late‐stage deflection, with a clear terminal change in direction when η>90%. Despite their geometric proximity, the two corner types diverge markedly near closure, indicating that the second axial crease separates the terminal motion into two pathways: stable inward contraction and deflection‐prone turnback. Accordingly, the second axial crease acts as a kinematic boundary in the folding of the FO–BW hybrid mode; nodes farther from this crease exhibit stronger late‐stage deflection, most evident at η>90%.

Figure 4E plots the angles $\theta_{8}$ and $\theta_{9}$—defined as the angles between the x‐axis and the lines $Op_{8}$ and $Op_{9}$ in Figure 4D—as a function of the folding ratio η (0%–100%). $\theta_{8}$ increases monotonically with η; it grows approximately linearly up to η=60% and then accelerates toward closure. $\theta_{8}$ starts at $59.04^{\circ }$ and reaches $162.67^{\circ }$, yielding a net anticlockwise rotation of $103.63^{\circ }$. In contrast, $\theta_{9}$ follows a similar increasing trend over most of the folding process but drops sharply near closure; the decrease becomes markedly steeper within η=99.6%–100%, indicating a strongly nonlinear response. $\theta_{9}$ begins at $45.00^{\circ }$, peaks at $115.73^{\circ }$ midway, and ends at $73.04^{\circ }$, corresponding to $70.73^{\circ }$ of anticlockwise accumulation followed by $52.67^{\circ }$ of clockwise rollback. This bidirectional angular evolution of $p_{9}$ demonstrates that the FO–BW hybrid mode retains the BW‐region bidirectional winding signature and indicates partial self‐unwinding via sequential rotations of opposite sign.

#### 2.3.3 Folding‐Induced Bidirectional Winding Characteristics of the BW‐FO Hybrid Mode

A 0.2‐mm‐thick paperboard sample in the BW–FO hybrid mode is fabricated, and its bidirectional winding behavior is examined experimentally. The folding ratio η is used to quantify folding progress, and θ is defined as the acute dihedral angle between creases $p_{1}p_{2}$ and $p_{1}p_{37}$.

**FIGURE 5:**
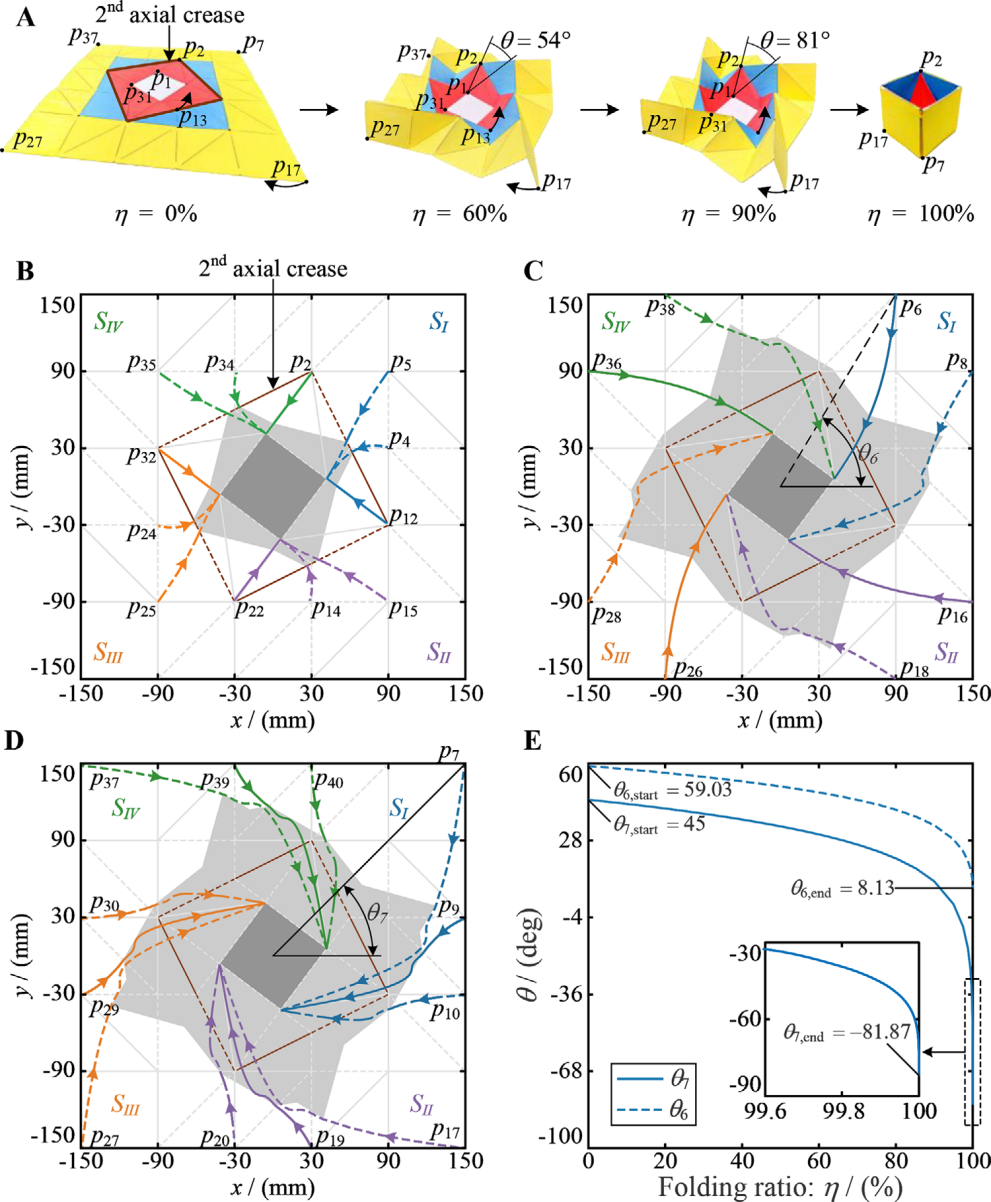
Folding‐Induced bidirectional winding characteristics of the BW–FO hybrid origami structure. (A) Folding process of a paperboard prototype (at representative configurations η=0%, 60%, 90%, and 100%). (B) Projection of the trajectories of nodes at the BW–outer‐FO interface onto the xy plane. (C)‐(D) Projection of the trajectories of the mid‐edge outer‐boundary nodes onto the xy plane, showing predominantly clockwise contraction with a pronounced late‐stage turning fluctuation that intensifies with increasing distance from the second axial crease, indicating that the outer FO‐mode region strengthens the clockwise bias. In (B)–(D), the light‐ and dark‐gray shaded regions denote the projected areas in the xy plane enclosed by the selected nodes at η=90% and 100%, respectively. (E) Angular metrics $\theta_{6}$ and $\theta_{7}$ for corners $p_{6}$ and $p_{7}$ as functions of η, showing an accelerated late‐stage decrease that highlights the amplification effect induced by the outer FO region.

Figure 5A shows the structural evolution at η=0%, 60%, 90%, and 100%. Compared with the FO–BW hybrid mode, which features pronounced rotation of axial‐crease nodes, the BW–FO hybrid mode exhibits boundary‐dominated motion. At η=60%, $p_{13}$ shows only a slight anticlockwise deflection, whereas $p_{17}$ already undergoes a distinct clockwise rotation. With increasing η, the clockwise rotation of $p_{17}$ accumulates and progressively dominates the boundary motion. The contrast between η=90% and η=100% reveals a rapid terminal tightening, with the apparent “acceleration” primarily associated with abrupt boundary twisting: the shrinking envelope volume coincides with localized, fast clockwise rotation of the boundary nodes. These observations indicate that the outer FO region imposes a stronger directional constraint on the boundary, thereby reinforcing the clockwise rotation of boundary nodes.

A theoretical model is introduced here to further examine the BW–FO hybrid mode. The incremental displacements of nodes $p_{2}$ and $p_{6}$ along the x‐direction, denoted as $dx_{2}$ and $dx_{6}$, respectively, are selected as representative generalized coordinates. A fixed proportional constraint, $dx_{6}=kdx_{2}$, is imposed throughout the folding process, thereby reducing the motion to a single‐parameter path fully parameterized by the folding ratio η. The related formulas for calculating the node coordinates are provided in Supporting Information S8. The projected trajectories of the selected nodes in the xy plane under the BW–FO hybrid mode are shown in Figure 5B–D. Consistent with Figure 4B–D, the projected areas in the xy plane of the origami regions enclosed by these nodes at η=90% and 100% are shaded in light and dark gray, respectively, in Figure 5B–D. The detailed construction procedure is provided in Figure S2.

Figure 5B plots the xy‐plane projections of node trajectories at the interface between the inner BW region and the outer FO region. Nodes $p_{2}$, $p_{12}$, $p_{22}$, and $p_{32}$ rotate about a fixed axis; accordingly, their projected trajectories are linear. Nodes $p_{4}$, $p_{14}$, $p_{24}$, and $p_{34}$ lie close to the second axial crease and show only weak clockwise entrainment, so their trajectories remain predominantly anticlockwise. By contrast, nodes $p_{5}$, $p_{15}$, $p_{25}$, and $p_{35}$ exhibit a directional switch, with a slight anticlockwise bias early and a mild clockwise trend late. Collectively, these trajectory types indicate that the second axial crease in the inner BW region biases the rotation direction of the interface nodes; the reversal remains subtle owing to their limited translational excursions and small accumulated angular displacement.

Figure 5C–D depicts the trajectories of the outer‐boundary nodes in the BW–FO hybrid mode. The boundary contracts predominantly clockwise, indicating that the outer FO region transmits and amplifies the clockwise tendency induced by the second axial crease toward the boundary. In sector $S_{I}$, node $p_{6}$ shows stronger kinematic coupling with $p_{2}$; its trajectory therefore approaches a circular arc with minimal directional fluctuation. By contrast, the remaining boundary nodes exhibit two pronounced turning fluctuations within the shaded region, and these fluctuations intensify with increasing distance from the second axial crease. These results suggest that the outer FO region reinforces clockwise inward contraction while amplifying nonuniform boundary‐node motion.

The boundary‐motion disparity is further quantified by comparing two adjacent nodes, $p_{6}$ and $p_{7}$, which exhibit clearly distinct trajectory signatures. Figure 5E plots the angular metrics $\theta_{6}$ and $\theta_{7}$ for $p_{6}$ and $p_{7}$ in Figure 5C–D as functions of the folding ratio η (0%–100%), where $\theta_{i}$ denotes the angle between $\vec{Op_{i}}$ and the x‐axis. The initial values of $\theta_{6}$ and $\theta_{7}$ are $59.03^{\circ }$ and $45.00^{\circ }$, respectively. With increasing η, both angles decrease overall, indicating clockwise inward contraction. $\theta_{6}$ decreases smoothly and terminates at $8.13^{\circ }$, corresponding to a net change of $50.90^{\circ }$. $\theta_{7}$ follows a similar trend over most of the folding process, whereas the final interval η=99.6%–100% shows an abrupt drop, with $\theta_{7}$ decreasing from $-32.66^{\circ }$ to $-81.87^{\circ }$; the net change over the full process is $126.82^{\circ }$. The late‐stage steepening highlights the enhanced clockwise‐rotation bias induced by the outer FO‐mode region, which becomes most pronounced near closure.

### 2.4 Bidirectional‐Winding Multilayer Origami Structure for Programmable Modular Expansion

**FIGURE 6:**
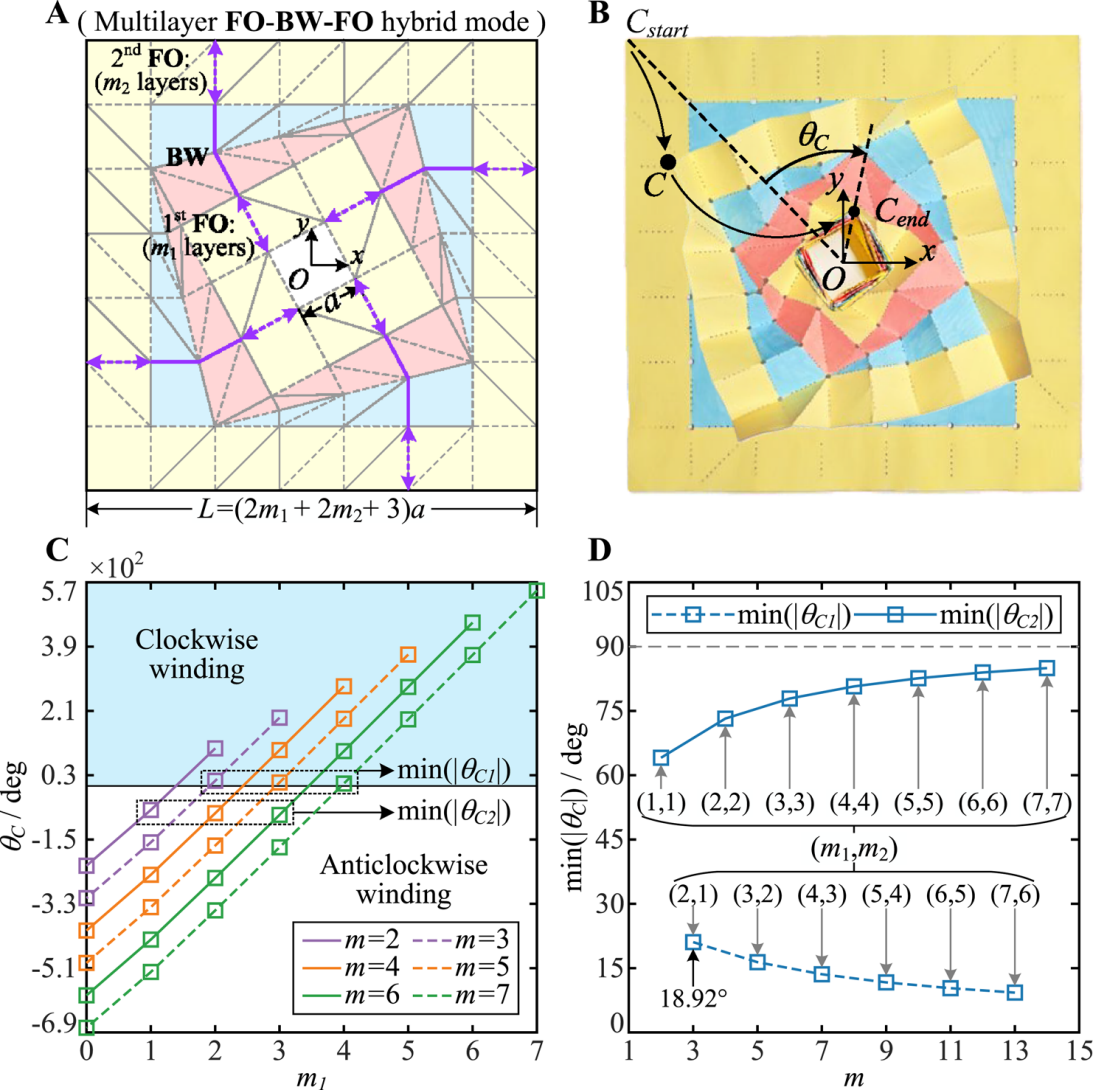
Bidirectional winding characteristics of Multilayer Hybrid Modes for programmable modular expansion. (A) Geometry schematic of the bidirectional‐winding multilayer origami structure. (B) Configuration evolution of the $\left(m_{1},m_{2}\right)=\left(1,1\right)$ design in the deployed, partially folded, and fully folded states. (C) Winding angle $\theta_{C}$ for different $\left(m_{1},m_{2}\right)$ pairs at a fixed total layer number $m=m_{1}+m_{2}$ (m=2,3,…,7); the horizontal axis is $m_{1}$ (with $m_{2}=m-m_{1}$). (D) Evolution of the minimum winding angles $\min(|\theta_{C1}|)$ and $\min(|\theta_{C2}|)$ for m=2,3,…,14.

Figure 6A schematically illustrates the bidirectional‐winding multilayer origami, establishing programmable modular expansion. This mode integrates a BW intermediate layer with inner and outer FO layers, forming a multilayer module. The inner FO layer contains $m_{1}$ layers, the outer FO layer contains $m_{2}$ layers, and the central hub has a side length a. The expansion scale is quantified by the outer‐boundary side length L, defined as $L=(2m_{1}+2m_{2}+3)a$. With a fixed, L is determined by $m_{1}$ and $m_{2}$; increasing either parameter enlarges L. Accordingly, multilayer FO–BW–FO hybrid configurations are indexed by $\left(m_{1},m_{2}\right)$, providing a direct mapping between layer numbers and structural scale to support programmable modular expansion design.

Figure 6B shows the configuration evolution of the bidirectional‐winding multilayer origami with $\left(m_{1},m_{2}\right)=\left(1,1\right)$ in three representative states: fully deployed, partially folded, and fully folded. During folding, corner C moves from $C_{\mathrm{start}}$ to $C_{\mathrm{end}}$. The bidirectional winding behavior is reflected in the rotation direction and magnitude of this corner motion; the winding level is therefore characterized by the winding angle $\theta_{C}$, defined as the rotation angle from the vector $\vec{OC_{\mathrm{start}}}$ to the vector $\vec{OC_{\mathrm{end}}}$ about the center O. The angle $\theta_{C}$ can be obtained from the two vectors as

(1) $$\theta_{C}=atan2\left(\left(\vec{OC_{\mathrm{start}}}\times \vec{OC_{\mathrm{end}}}\right)\cdot e_{z},\vec{OC_{\mathrm{start}}}\cdot \vec{OC_{\mathrm{end}}}\right)$$

where $e_{z}$ is the unit vector normal to the plane. A detailed explanation and illustrative example of the atan2 function are provided in Supporting Information S9.

The influence of different $\left(m_{1},m_{2}\right)$ combinations on $\theta_{C}$ is evaluated by introducing the total layer number $m=m_{1}+m_{2}$. This $\left(m_{1},m_{2}\right)$ design space links layer allocation to winding response, providing a programmable route for modular‐scale expansion. Figure 6C plots $\theta_{C}$ for m=2,3,…,7 with anticlockwise rotation defined as positive. The horizontal axis is $m_{1}$, ranging from 0 to m, with $m_{2}=m-m_{1}$. At $m_{1}=0$, $\theta_{C}<0^{\circ }$ across all m, indicating a clockwise folding tendency of corner C, consistent with the observations above. As $m_{1}$ increases, $\theta_{C}$ increases linearly with a slope independent of m, rendering the curves parallel. The sign of $\theta_{C}$ indicates rotation direction, whereas the winding magnitude is given by $|\theta_{C}|$. The monotonic dependence on $m_{1}$ implies that each fixed m admits a unique combination within $m_{1}\in \left[0,m\right]$ that minimizes $|\theta_{C}|$, denoted as $\min(|\theta_{C}|)$. The minimizer yields $\theta_{C}<0^{\circ }$ for even m and $\theta_{C}>0^{\circ }$ for odd m. Accordingly, $\min(|\theta_{C}|)$ is denoted as $\min(|\theta_{C1}|)$ when m is odd and as $\min(|\theta_{C2}|)$ when m is even.

Figure 6D extends the analysis to larger m and summarizes the evolution of $\min(|\theta_{C1}|)$ and $\min(|\theta_{C2}|)$ for m=2,3,…,14, with the corresponding $\left(m_{1},m_{2}\right)$ pairs annotated. Specifically, $\min(|\theta_{C1}|)$ is attained at $\left(m_{1},m_{2}\right)=\left((m+1)/2,(m-1)/2\right)$, whereas $\min(|\theta_{C2}|)$ is attained at $\left(m_{1},m_{2}\right)=\left(m/2,m/2\right)$. For odd m, $\min(|\theta_{C1}|)$ decreases toward $0^{\circ }$ as m increases, with a progressively weaker rate of convergence; for even m, $\min(|\theta_{C2}|)$ increases toward $90^{\circ }$ as m increases, again with a progressively weaker rate of convergence. The limiting behavior of $\min(|\theta_{C1}|)$ and $\min(|\theta_{C2}|)$ is further visualized in Figure S3.

Figure 6C,D translates multilayer hybridization into a programmable design map, enabling modular expansion with predictable winding output. Under prescribed geometric constraints (e.g., L or a), selecting $\left(m_{1},m_{2}\right)$ directly tunes $\theta_{C}$, allowing the expanded module to meet application‐specific requirements. As further shown in Figure S4, assigning the layer‐partition pair $\left(m_{1},m_{2}\right)$ generates a discrete set of attainable winding angles without changing the central hub or outer boundary size, thereby establishing a rich library of winding‐angle candidates for programmable deployment design. For low‐shock deployment in large‐area space structures (e.g., membrane antennas and solar sails), $\left(m_{1},m_{2}\right)$ can be chosen to minimize $|\theta_{C}|$ and reduce winding‐induced disturbance. For systems requiring coordinated motion (e.g., automotive sunshades and petal‐like retractable roofs), $\left(m_{1},m_{2}\right)$ can be selected to deliver a target $\theta_{C}$, enabling kinematic synchronization across coupled components.

### 2.5 A Novel Membrane Deployable Structure Based on Bidirectional‐Winding Multilayer Origami

#### 2.5.1 Structural Design of the Membrane Deployable Structure

**FIGURE 7:**
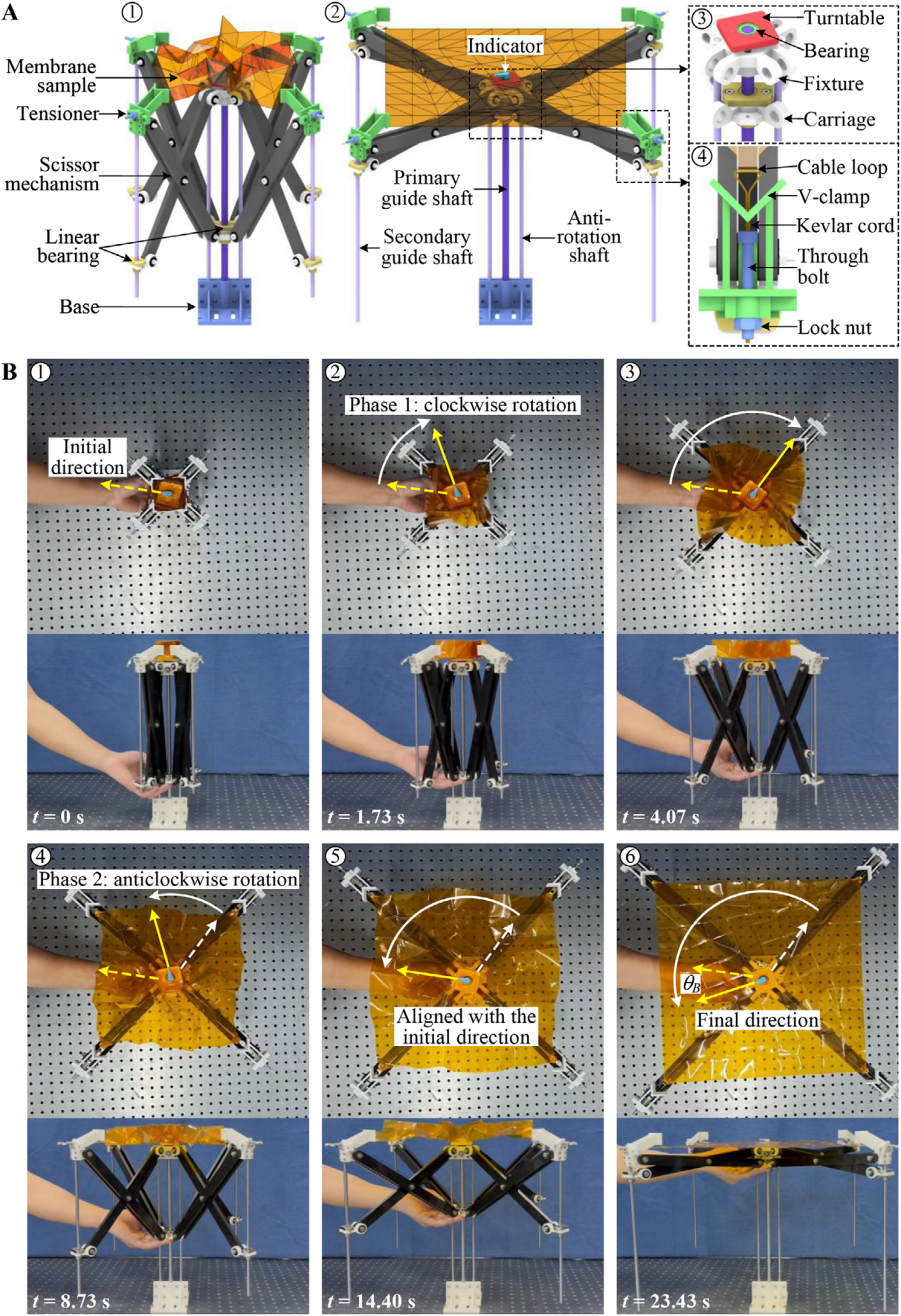
A novel membrane deployable structure based on bidirectional‐winding multilayer origami. (A) Structural design of the deployment apparatus, which consists of four scissor mechanisms, four tensioners, multiple guide shafts, and a turntable; the indicator denotes the rotation direction. (B) Bidirectional winding deployment of the membrane sample. Synchronized top and front views at key instants: ① initial clamped state; ②–③ Phase I, clockwise rotation with progressive deceleration; ④–⑤ Phase II, anticlockwise rotation with an approximate mid‐stroke zero crossing; ⑥ fully deployed state, showing additional net anticlockwise rotation in the final stage.

Membrane deployable structures are a widely used and efficient structural solution in civil and aerospace engineering. A novel membrane deployable structure is developed from the proposed bidirectional‐winding multilayer origami. The origami configuration with $\left(m_{1},m_{2}\right)=\left(2,1\right)$ and a=40mm is selected, and a membrane sample is fabricated from a 50μm‐thick polyimide membrane. A scissor‐mechanism‐based deployment system is implemented, enabling controlled actuation and synchronized deployment. Figure 7A ① shows the complete device, comprising the membrane sample, four scissor‐mechanism assemblies, a tensioning unit, guide rails, a base, and auxiliary components.

As shown in Figure 7A ②, the guide‐shaft system constrains the orientations of the moving components and is essential for reliable operation. The primary guide shaft governs the kinematics: a fixture–carriage assembly couples the four scissor‐mechanism units into a single translational degree of freedom, ensuring synchronous motion. The fixture is rigidly attached to the primary guide shaft, and the carriage slides linearly along it (Figure 7A ③). Two anti‐rotation shafts are placed on both sides of the primary guide shaft to suppress carriage rotation about the shaft axis. Additionally, four secondary guide shafts ensure that the top tensioners remain level and translate along a straight path throughout deployment.

During deployment, the membrane sample requires an effective rotation of magnitude $|\theta_{C}|$: the four corners translate along straight lines, while the geometry induces a net global rotation. A turntable (Figure 7A ③) accommodates this mismatch and prevents parasitic torsion at the corner connections. Sized to the central hub, the turntable is mounted on the primary guide shaft via a bearing, enabling self‐aligned rotation about the center. An indicator (Figure 7A ②) at the sample center visualizes the rotation direction.

The tensioner is shown in Figure 7A ④. Cable loops are installed at the four membrane corners and connected to the tensioner via Kevlar cords; tension is continuously adjustable via a through‐bolt and lock nut. A V‐shaped clamp is integrated into the base, constraining and securing the stowed size when the membrane is fully folded.

#### 2.5.2 Experimental Demonstration of Bidirectional‐Winding Behavior

After the membrane sample is mounted, the apparatus is fixed on an optical table, and the carriage is driven at a constant speed to deploy the membrane. The process is recorded synchronously from the top and front views (see Video S1), and the observations are shown in Figure 7B.

As shown in Figure 7B ①, the membrane sample is clamped in the initial state, and a yellow dashed arrow marks the indicator's initial direction. As the carriage moves upward, the membrane sample enters Phase I (clockwise rotation). At t=1.73s (Figure 7B ②), the carriage advances only a short distance, whereas the indicator already undergoes a substantial clockwise rotation, reaching nearly half of the Phase‐I rotation. At t=4.07s (Figure 7B ③), the indicator reaches the terminal orientation of the clockwise phase. Although the angular changes from ①→② and ②→③ are comparable, the latter interval lasts 0.61s longer, indicating a lower mean angular velocity and a progressive slowdown as deployment proceeds. In addition, the carriage displacement from the initial position to the end of Phase I accounts for only 10% of the total deployment stroke, corroborating the earlier finding that kinematic variation becomes pronounced once η>90%.

The membrane sample then enters Phase II (anticlockwise rotation). At t=8.73s (Figure 7B ④), the indicator returns to the same direction as in ②; the ③→④ interval lasts 4.66s, substantially longer than ②→③, indicating a lower mean angular velocity in Phase II. At t=14.40s (Figure 7B ⑤), the indicator aligns with its initial direction, showing that the Phase‐II anticlockwise rotation largely offsets the Phase‐I clockwise rotation and drives the effective relative rotation close to zero. The carriage is near mid‐stroke, indicating that this zero‐crossing occurs around the stroke midpoint. At t=23.43s (Figure 7B ⑥), the membrane sample is fully deployed. The indicator remains offset from its initial direction, with the initial direction lying clockwise of the final one, indicating an additional net anticlockwise rotation over the second half of the stroke.

### 2.6 Experimental Testing of the Bidirectional‐Winding Behavior of the Membrane Deployable Structure

#### 2.6.1 Photogrammetry‐Based Setup for Discrete 3D Coordinate Measurement

**FIGURE 8:**
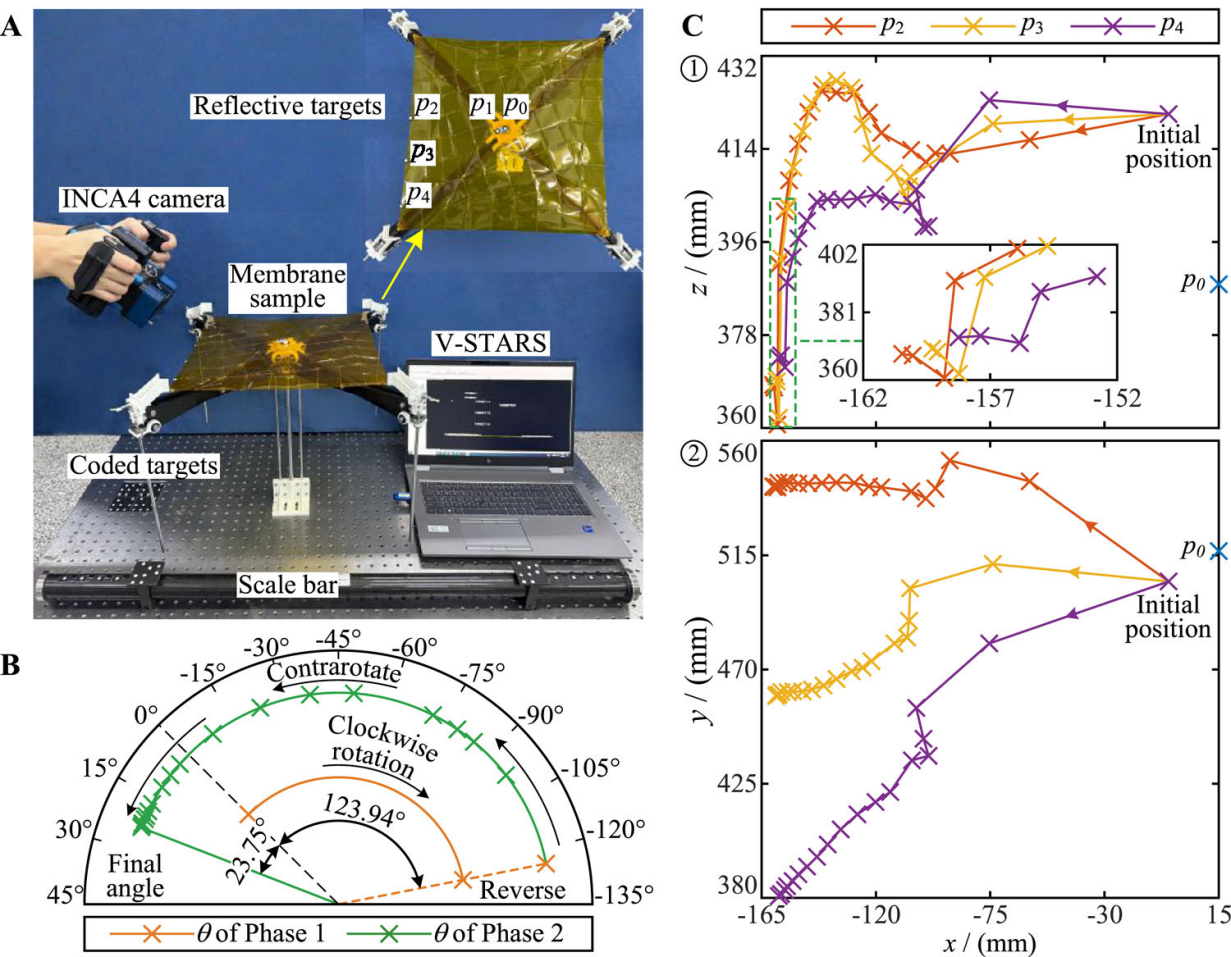
Experimental Testing of the Bidirectional‐Winding Behavior of the Membrane Deployable Structure. (A) Experimental setup for photogrammetric measurement. (B) Evolution of the indicator azimuth angle θ along the deployment stroke (with the initial direction defined as $0^{\circ }$), capturing the two‐phase rotation—clockwise in Phase I and anticlockwise in Phase II—as well as the net additional anticlockwise rotation in the fully deployed state. (C) Deployment trajectories of representative nodes $p_{2}$, $p_{3}$, and $p_{4}$: ① xz‐plane projection (out‐of‐plane height evolution), and ② xy‐plane projection (in‐plane trajectories), revealing the clockwise‐to‐anticlockwise phase transition and the tension‐dominated late‐stage convergence toward an approximately coplanar configuration.

A quantitative characterization of the two‐phase rotation and the accumulated net anticlockwise rotation is obtained using the V‐STARS INCA4 industrial photogrammetry system. The deployment is sampled at discrete instants, and the indicator orientation and 3D motion of representative nodes are converted into 3D coordinate data (Figure 8A). Reflective targets $p_{0}$–$p_{4}$ are attached to the membrane and the indicator: $p_{0}$ and $p_{1}$ mark the base and tip of the pointer, defining its direction vector and azimuth; $p_{2}$, $p_{3}$, and $p_{4}$ are placed at representative crease vertices to capture deployment kinematics. Coded targets and a scale bar are placed on the optical table to calibrate the cameras and establish a global coordinate system.

Measurement repeatability and cross‐test comparability are ensured by using the carriage stroke as the control variable. The total stroke is divided into 20 preset positions, and the carriage is positioned and locked at each position with a support fixture, enabling a discrete “stroke–acquire–advance” workflow. The apparatus and membrane attitude are constrained to maintain consistent step lengths and repeatable measurement geometry.

During measurement, the INCA4 camera is handheld and moved around the sample in a top‐down configuration, acquiring multiple overlapping images from different viewpoints. The images are imported into V‐STARS for camera calibration, spatial intersection, and global coordinate reconstruction. Reconstruction quality is verified using residuals, image‐point matching metrics, and scale‐bar errors, and the 3D coordinates of all targets are reported in a unified coordinate system.

#### 2.6.2 Quantitative Evolution of the Indicator Azimuth

The azimuth of the indicator is obtained from the global xy‐plane projection of the direction vector $\vec{p_{0}p_{1}}$ and is denoted as θ (Figure 8B). The initial pointing direction serves as the $0^{\circ }$ reference. Phase I occupies only a short carriage stroke; uniform stroke sampling therefore places the second data point at the Phase‐I endpoint, yielding $\theta =123.94^{\circ }$. The third data point shows a negligible change, indicating a brief rotational pause and direction reversal over this interval. The remaining points capture Phase II (anticlockwise rotation), and the progressively smaller Δθ between adjacent points indicates continued deceleration with carriage advance, consistent with the fast‐then‐slow trend in Figure 7B ①–③. The azimuth angle ends at $23.75^{\circ }$, corresponding to the net additional anticlockwise rotation in the fully deployed state (Figure 7B ⑥).

The theoretical winding angle for $\left(m_{1},m_{2}\right)=\left(2,1\right)$ in Figure 6D is $|\theta_{C1}|=18.92^{\circ }$. Using the experimental terminal angle as the reference, the relative deviation with respect to the experimental value is 20.3%. Two factors account for this discrepancy. (1) Finite membrane thickness becomes non‐negligible after compaction, shifting the effective geometry and biasing θ; this effect reduces with larger geometric scale or thinner membrane. (2) Manual fabrication tolerances (cutting, bonding, and positioning) accumulate in corner and crease geometry; automated fabrication and dedicated assembly jigs would reduce this error. Overall, Figure 8B confirms the two‐phase rotation—clockwise in Phase I and anticlockwise in Phase II—and demonstrates winding‐angle reduction via reverse rotation.

#### 2.6.3 3D Trajectory Analysis of Representative Nodes

The 3D coordinates of $p_{2}$, $p_{3}$, and $p_{4}$ are plotted as deployment trajectories in Figure 8C. Figure 8C ① shows the xz‐plane projections and Figure 8C ② shows the xy‐plane projections. The three nodes start from nearly coincident positions and are therefore treated as a single initial point, consistent with the theoretical assumption. In Figure 8C ①, the second sampled frame shows an ordered height distribution, $z_{p_{2}}<z_{p_{3}}<z_{p_{4}}$; $p_{4}$ lies above the initial point, whereas $p_{2}$ and $p_{3}$ lie below it, indicating an initial lift of the corner region. The heights then decrease, with the drop magnitude ranked as $p_{4}>p_{3}>p_{2}$, indicating reduced out‐of‐plane support after the clockwise phase and gravity‐dominated sag. The ranking follows radial distance from $p_{0}$ (from $p_{2}$ to $p_{4}$), indicating weaker crease constraint transmission outward and thus larger out‐of‐plane deflection. During the anticlockwise phase, the heights show a rise–fall response, indicating partial recovery of traction support and a non‐monotonic geometric effect. Near full deployment, all three nodes rise slightly from their minima and converge in height, indicating that increasing membrane tension suppresses sag and drives the points toward an approximately coplanar, fully tensioned state.

In Figure 8C ②, the trajectories of the three nodes nearly collapse onto a single curve, except for the first three sampled frames. These early points create an apparent in‐plane jump, arising from the rotation–stroke mismatch in Phase I: a large angular change occurs over a short carriage travel, and uniform stroke sampling compresses Phase I into only a few frames. Phase II exhibits progressively smoother trajectories, indicating that in‐plane motion becomes crease‐constrained and approaches a smooth unfolding driven by corner translation.

### 2.7 Winding‐Angle Programmability Across Origami Structures

**FIGURE 9:**
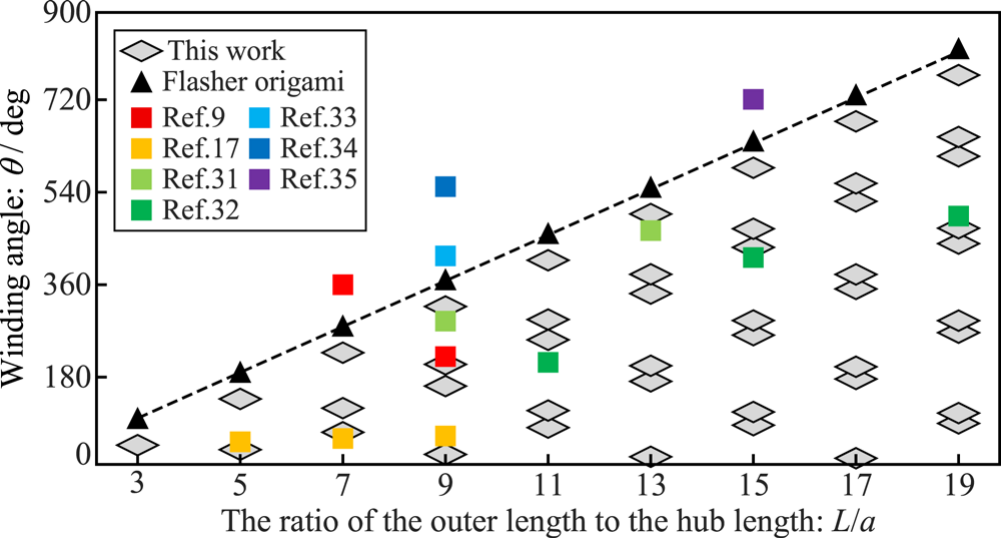
Comparison of winding‐angle programmability among different origami structures.

A comparison is conducted between the bidirectional‐winding multilayer origami structure and multiple representative winding origami configurations. The benchmark set includes the canonical Flasher origami and seven winding origami configurations reported in [10, 24, 39, 40, 41, 42, 43], denoted Ref. 9, Ref. 17, and Ref. 31–Ref. 35. The results are summarized in Figure 9. A unified geometric scale is introduced by defining the horizontal axis as L/a, the ratio of the outer‐boundary side length to the central‐hub side length. The winding angles of all configurations are reported for L/a=3,5,…,19.

Figure S5 shows three representative structural configurations corresponding to L/a=3,5, and 7; configurations with L/a=9,11,…,19 can be generated by extending this sequence. In Flasher origami, the winding angle is independent of the absolute values of L and a, but increases linearly with the ratio L/a, increasing by $90^{\circ }$ for every two‐unit increase in L/a. Equivalently, every two‐unit increase in L/a produces a $90^{\circ }$ increase in the winding angle. Ref. 9, Ref. 17, and Ref. 31–Ref. 32 exhibit two or three distinct winding angles, whereas Ref. 33–Ref. 35 show only a single winding angle. Some structures retain a relatively small winding angle throughout folding (e.g., Ref. 17), whereas others exhibit a Flasher‐like increasing trend with L/a (e.g., Ref. 31 and Ref. 32). Still others generate winding angles even larger than those of Flasher origami at the same L/a (e.g., Ref. 34 and Ref. 35). Despite these differences, a shared feature emerges: at a fixed L/a—that is, under a prescribed overall size and unit scale—each structure admits only one folding mode and one unique winding angle, indicating that its kinematics are geometrically constrained.

By contrast, the bidirectional‐winding multilayer origami structure does not exhibit this constraint. Across all L/a, hybrid integration yields an expandable set of attainable winding angles: at a fixed L/a, different $\left(m_{1},m_{2}\right)$ combinations generate multiple winding angles, demonstrating programmable winding‐angle tunability. Increasing L/a further enlarges both the number of attainable angles and the coverage range. For each L/a, the evolution of the minimum winding angle follows the trend in Figure 6D. Distinct target angles can therefore be achieved at the same L/a by selecting appropriate $\left(m_{1},m_{2}\right)$, enabling the winding angle to be matched on demand without changing the central‐hub or outer‐boundary scale. The attainable minimum winding angle also makes this structure well‐suited for applications with strict winding‐stroke limits or requiring reduced deployment shock.

## 3 Conclusion

Origami‐inspired winding deployable structures offer compelling advantages for ultralight, large‐scale spacecraft, combining low mass, high packaging efficiency, and high stiffness. Discretized winding angles and strong scale–angle coupling remain key limitations of existing winding origami, enforcing unavoidable size–function trade‐offs. This work presents a bio‐inspired origami system with bidirectional winding, enabling programmable, modular reconfiguration and scalable extension.

First, inspired by the second axial crease of earwig hindwings, key crease features are mapped into a parameterized and controllable crease‐line network, yielding an origami unit that exhibits second axial crease. Circumferential assembly of this unit generates a bidirectionally winding origami structure, and analyzes and experiments confirm opposite winding tendencies between the inner‐wing and outer‐wing regions during folding and deployment.

Next, hybridization with Flasher origami produces two coupled modes by integrating Flasher origami with the inner‐wing and outer‐wing regions, respectively. Both modes preserve bidirectional winding while inheriting the scalability of Flasher origami; moreover, Flasher origami amplifies the motion range of the coupled region.

Building on these results, the Bidirectional‐Winding Multilayer Origami Structure is developed, demonstrating that tuning the layer numbers of the two Flasher modes converts the winding angle into a designable and adjustable geometric parameter. By assigning the layer numbers without changing the central hub or outer boundary size, this architecture generates a discrete set of attainable winding angles. Within this discrete design space, a rich library of winding‐angle candidates is formed, providing multiple options for programmable deployment design.

Finally, a membrane deployment mechanism based on the Bidirectional‐Winding Multilayer Origami Structure demonstrates a bidirectional winding strategy involving sequential clockwise and counterclockwise rotations. Experiments track the coordinates of key nodes during deployment, showing that bidirectional winding reduces the required rotation and improves deployment efficiency. Overall, this work establishes a programmable and scalable platform for large‐area forced‐deployment systems, with broad potential in space mechanisms and architectural structures.

## 4 Experimental Section

### 4.1 Sample Preparation

The experimental specimen adopts the bidirectional‐winding multilayer origami structure with $\left(m_{1},m_{2}\right)=\left(2,1\right)$ and is fabricated from a 50μm‐thick polyimide membrane, with a central hub side length of 40mm. Plastic strain at crease lines would compromise folding–deployment performance; therefore, crease lines are implemented by precision cutting along the predefined origami pattern rather than by scoring. The resulting square and triangular facets are bonded using 38μm‐thick PET tape; cutting and bonding are carried out concurrently to improve geometric alignment accuracy. Corner‐driven tensile actuation is enabled by reserving attachment points at the four corners of the device. Aluminum tubes with a diameter of 0.8mm are fixed at the corner locations using 50μm‐thick polyimide tape, which distributes tensile loads and enhances the tensile capacity of the corner regions. A Kevlar cord is then threaded through each aluminum tube, allowing external tension to be transmitted stably to the membrane corners and thereby drive deployment.

### 4.2 Deployment Apparatus Design

A dedicated deployable structure is designed, as shown in Figure 7A. The system is built around a scissor mechanism and incorporates a guide‐rail assembly that constrains the motion posture, ensuring stable and controllable deployment. The central primary guide shaft couples the four scissor units into a single translational degree of freedom through a fixture–carriage assembly, thereby enforcing synchronized motion. Two anti‐rotation shafts are positioned symmetrically on both sides of the primary guide shaft, thereby preventing parasitic rotation during synchronization by suppressing carriage rotation about the shaft axis. Secondary guide shafts constrain the top tensioning module, maintaining horizontal linear translation throughout deployment. A turntable is mounted on the primary guide shaft via a bearing, which allows adaptive rotation of the membrane center and compensates for the relative rotation induced by geometric evolution. The tensioning module provides continuously adjustable tensile force through a through‐bolt and lock‐nut assembly, and a V‐shaped clamp integrated into the base limits and fixes the stowed dimension of the membrane sample in the folded state. In addition, an indicator (Figure 7A ②) is placed at the membrane center to visually record the rotation direction and angular variation of the turntable.

### 4.3 Experimental Setup

The bidirectional winding validation experiment is recorded from both top and front views, using 4K/60 fps video, and representative deployment states are extracted through frame‐by‐frame analysis. For quantitative evaluation, the deployment process of the membrane sample is discretely measured using an industrial photogrammetry system, with the setup shown in Figure 8A. Reflective targets are attached to both the membrane sample and the indicator: targets on the membrane capture the spatial motion of representative nodes during deployment, whereas targets on the indicator define the pointer direction vector and enable computation of its azimuth angle. Coded targets and a scale bar are arranged on the optical table to complete camera calibration and establish a global coordinate system. Measurement repeatability and cross‐test comparability are ensured by adopting the carriage stroke as the control variable. The total stroke is divided into 20 preset positions, and the carriage is positioned and locked at each location using a support fixture, forming a discrete “stroke–acquire–advance” workflow. During acquisition, the INCA4 camera is handheld and moved around the sample in a top‐down configuration, capturing multiple high‐overlap images from different viewpoints. The images are then imported into the industrial close‐range photogrammetry system to perform camera calibration, spatial intersection, and global coordinate‐system reconstruction. Reconstruction quality is verified through residuals, image‐point matching metrics, and scale‐bar errors, and the 3D coordinates of all targets are ultimately reported in a unified global coordinate frame.

## Conflicts of Interest

The authors declare no conflicts of interest.

## Supporting information


**Supporting File 1**: advs76652‐sup‐0001‐SuppMat.pdf.

**Supporting File 2;** advs76652‐sup‐0002‐VideoS1.mp4.

## Data Availability

Research data are not shared.
