# Supplementary material for: Bio‐Inspired Bidirectional Winding Origami With Programmable Modular Reconfigurability and Scalability
Source: Adv Sci (Weinh). 2026 Jul 20:e76652. Online ahead of print. doi: 10.1002/advs.76652 (PMC13383707; doi:10.1002/advs.76652)
Supplement: Supplementary file 1 — Supporting File 1: advs76652‐sup‐0001‐SuppMat.pdf. [file ADVS-9999-e76652-s001.pdf]

© 2026 Wiley-VCH

ADVANCED SCIENCE
Open Access

Supporting Information

Bio-Inspired Bidirectional Winding Origami with Programmable Modular Reconfigurability and Scalability

Wenyao Zhang, Chunlong Wang, Mingli Liu, Chuang Shi, Hongwei Guo, and Rongqiang Liu**

Figure S1

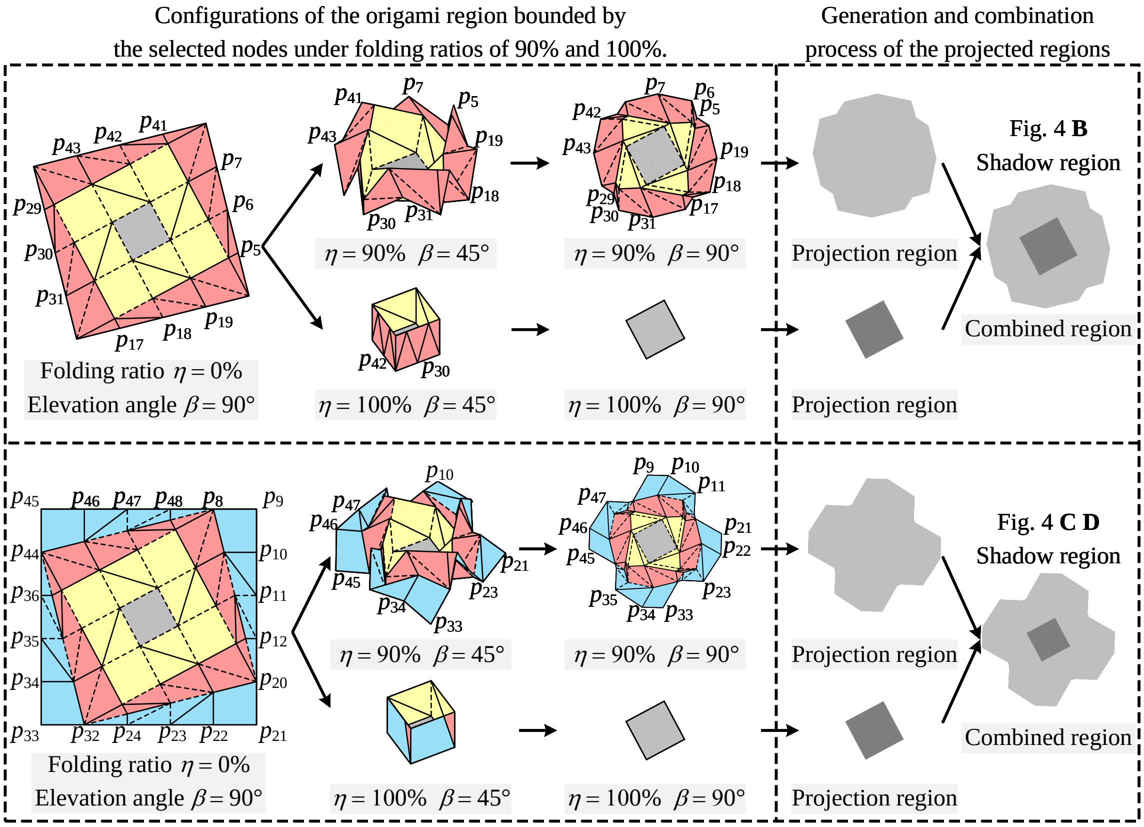

Figure S1: Schematic illustration of the generation of the shaded regions in Fig. 4(B)-(D). The origami region bounded by the selected nodes is projected onto the plane of the central hub along the direction corresponding to an elevation angle of 90° at folding ratios of 90% and 100%. The two projected regions are shown in light gray and dark gray, respectively, and are subsequently superimposed to form the combined shaded region.

Figure S2

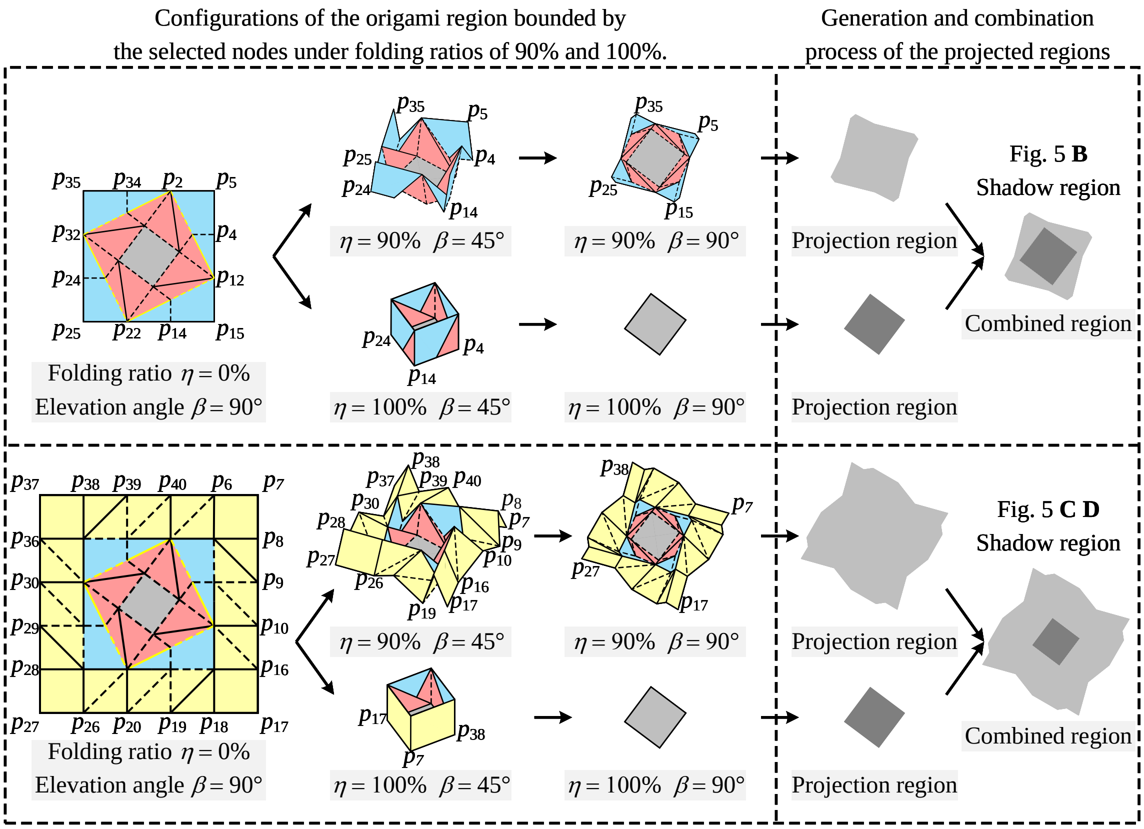

Figure S2: Schematic illustration of the generation of the shaded regions in Fig. 5(B)-(D). The origami region bounded by the selected nodes is projected onto the plane of the central hub along the direction corresponding to an elevation angle of 90° at folding ratios of 90% and 100%. The two projected regions are shown in light gray and dark gray, respectively, and are subsequently superimposed to form the combined shaded region.

Figure S3

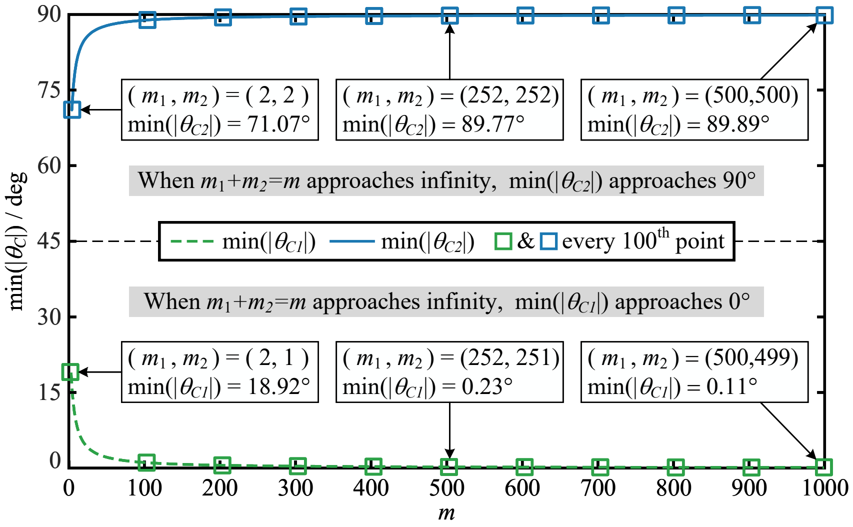

Figure S3: Limiting trend curves of $\min(|\theta_{C1}|)$ and $\min(|\theta_{C2}|)$. In the limiting case as m approaches infinity, $\min(|\theta_{C1}|)$ asymptotically approaches 0°, whereas $\min(|\theta_{C2}|)$ approaches 90°.

Figure S4

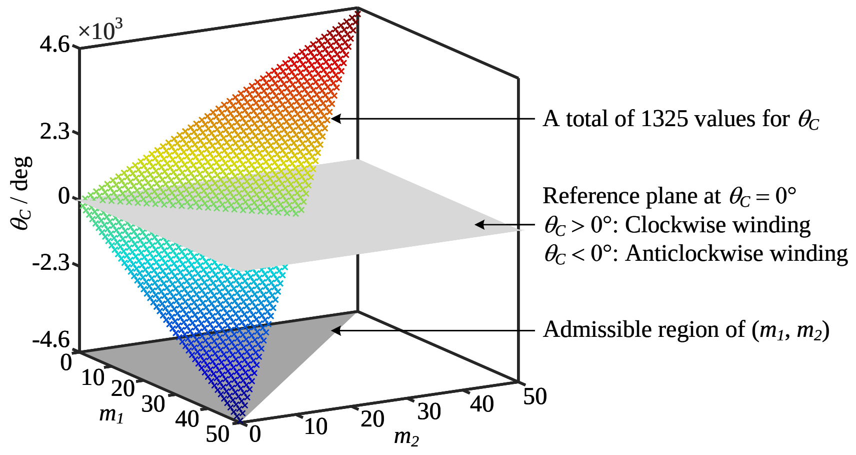

Figure S4: Distribution of the winding angle θ_C for all layer-partition pairs (m_1, m_2) with $m \leq 50$. Each marker "X" represents a calculated θ_C value, yielding a total of 1325 plotted values. The marker color denotes the magnitude of θ_C.

Figure S5

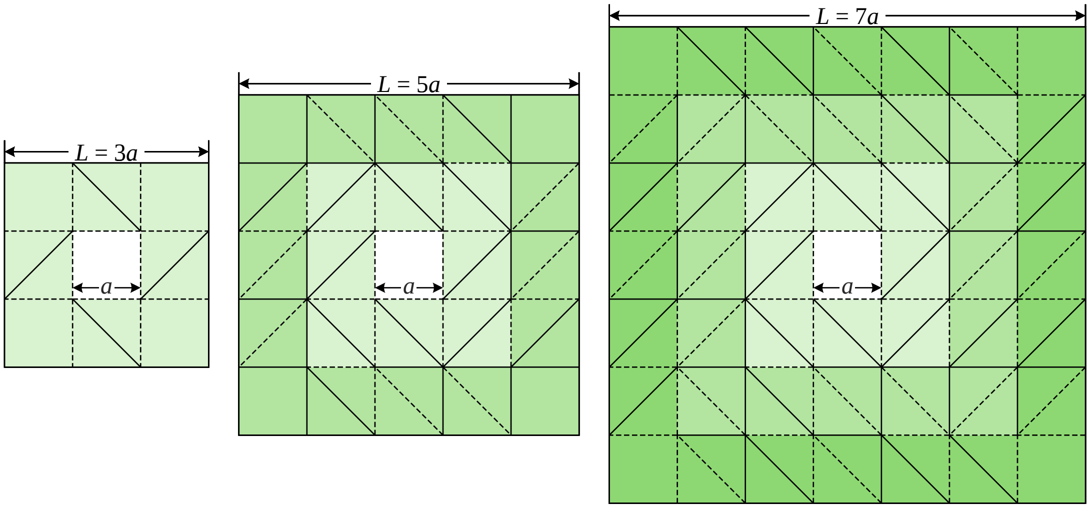

Figure S5: Configuration generation of Flasher origami in Fig. 9. Three representative structural configurations corresponding to $L/a = 3$, 5, and 7 are shown, while configurations with $L/a = 9, 11, \ldots, 19$ can be generated by extending this sequence.

S6. Nodal Coordinate Generation for the Bidirectional-Winding Origami Structure

As shown in Figure 1B of the main text, the bidirectional-winding origami structure is generated from a basic double axial-folding origami unit. The unit contains two representative crease-line subunits, namely a four-crease unit and a five-crease unit. A Cartesian coordinate system is established with the center of the square $p_1p_6p_{11}p_{16}$ as the origin O, and the x-axis is set to be parallel to edge p_1p_6.

During folding, nodes p_1, p_6, p_{11}, and p_{16} remain fixed in the xy-plane and serve as reference nodes. Their coordinates are prescribed as

$$\begin{cases} \mathbf{p}_1 = (-a/2,\ a/2,\ 0)^{\mathrm{T}}, \\ \mathbf{p}_6 = (a/2,\ a/2,\ 0)^{\mathrm{T}}, \\ \mathbf{p}_{11} = (a/2,\ -a/2,\ 0)^{\mathrm{T}}, \\ \mathbf{p}_{16} = (-a/2,\ -a/2,\ 0)^{\mathrm{T}}. \end{cases} \tag{E1}$$

For any two connected nodes p_i and p_j, the rigid-facet constraint is written in the compact form

$$\|\mathbf{p}_i - \mathbf{p}_j\| = L_{i,j}, \tag{E2}$$

where $\mathbf{p}_i = (x_i, y_i, z_i)^{\mathrm{T}}$ is the coordinate vector of node p_i, and $L_{i,j}$ denotes the distance between nodes p_i and p_j.

The coordinates of nodes p_2 and p_7 can be parameterized by setting $y_2 = t$:

$$\begin{cases} \mathbf{p}_2 = (-a/2,\ t,\ z(t))^{\mathrm{T}}, \\ \mathbf{p}_7 = (t,\ a/2,\ z(t))^{\mathrm{T}}, \end{cases} \qquad z(t) = \frac{\sqrt{3a^2 + 4at - 4t^2}}{2}. \tag{E3}$$

where t is introduced as the folding parameter of the bidirectional-winding origami unit. It denotes the prescribed y-coordinate of node p_2 in the reference unit. As t varies, the out-of-plane coordinate $z(t)$ of nodes p_2 and p_7 changes accordingly, thereby parameterizing the folding configuration of the bidirectional-winding unit. The admissible range of t is determined by the requirement that $z(t)$ remains real, together with the geometric constraints imposed by the rigid facets.

For compact expression, the three-distance intersection operator $T_s(\cdot)$ defined in Equation (E4) is introduced. If a target node p_A is determined by its distances to three known nodes p_B, p_C, and p_D, then

$$\mathbf{p}_A = T_s\left(\mathbf{p}_B, \mathbf{p}_C, \mathbf{p}_D; L_{A,B}, L_{A,C}, L_{A,D}\right), \tag{E4}$$

which means that $\mathbf{p}_A$ satisfies

$$\begin{cases} \|\mathbf{p}_A - \mathbf{p}_B\| = L_{A,B}, \\ \|\mathbf{p}_A - \mathbf{p}_C\| = L_{A,C}, \\ \|\mathbf{p}_A - \mathbf{p}_D\| = L_{A,D}. \end{cases} \tag{E5}$$

Here, $s = \pm 1$ denotes the selected folding branch, which is determined by the mountain-valley assignment of the corresponding crease.

The coordinate of node p_A is obtained by solving the three distance equations in Equation (E5), and the appropriate solution branch is selected according to the mountain-valley assignment of the corresponding crease. Using this operator, the remaining nodes of the bidirectional-winding unit can be expressed as

$$\begin{cases} \mathbf{p}_5 = T_s\left(\mathbf{p}_2, \mathbf{p}_6, \mathbf{p}_7; L_{5,2}, L_{5,6}, L_{5,7}\right), \\ \mathbf{p}_4 = T_s\left(\mathbf{p}_2, \mathbf{p}_5, \mathbf{p}_7; L_{4,2}, L_{4,5}, L_{4,7}\right), \\ \mathbf{p}_3 = \mathbf{p}_2 - 2\left(\mathbf{p}_5 - \mathbf{p}_4\right). \end{cases} \tag{E6}$$

The complete bidirectional-winding origami structure is obtained by rotational arraying of the derived unit-cell coordinates about the central axis. The rotation matrix of the kth sector is

$$\mathbf{R}_k = \begin{bmatrix} \cos(90^\circ k) & -\sin(90^\circ k) & 0 \\ \sin(90^\circ k) & \cos(90^\circ k) & 0 \\ 0 & 0 & 1 \end{bmatrix}, \qquad k = 0, 1, 2, 3. \tag{E7}$$

Thus, the corresponding node in the kth sector is obtained by

$$\mathbf{p}_i^{(k)} = \mathbf{R}_k \mathbf{p}_i. \tag{E8}$$

Through Equations (E1)-(E8), all nodal coordinates of the bidirectional-winding origami structure can be determined.

S7. Nodal Coordinate Generation for the FO-BW Hybrid Mode

The FO-BW hybrid mode is constructed by coupling a Flasher origami (FO) sector with a bidirectional-winding origami (BW) sector. A representative sector is first defined by geometric constraints, and the complete structure is then generated through rotational arraying. The FO-BW hybrid mode contains 48 nodes, which are divided into four identical sectors, with each sector containing 12 nodes.

The node-index mapping for the rotational array is defined as

$$I_k(i) = 1 + \mathrm{mod}(i + 12k - 1, 48), \qquad i = 1, 2, \ldots, 12, \quad k = 0, 1, 2, 3. \tag{E9}$$

The corresponding node in the kth sector is obtained as

$$\mathbf{p}_{I_k(i)} = \mathbf{R}_k \mathbf{p}_i, \tag{E10}$$

where $\mathbf{R}_k$ is the rotation matrix defined in Equation (E7).

The geometric constraints of the reference sector are expressed by

$$\|\mathbf{p}_i - \mathbf{p}_j\| = L_{i,j}, \qquad (i,j) \in E_{\mathrm{FOBW},0}, \tag{E11}$$

where $E_{\mathrm{FOBW},0}$ denotes the crease-line set of the reference sector.

The initial nodes are prescribed as

$$\begin{cases} \mathbf{p}_1 = (a/2,\ a/2,\ 0)^{\mathrm{T}}, \\ \mathbf{p}_2 = \left(t_1,\ a/2,\ \sqrt{a^2 - (t_1 - a/2)^2}\right)^{\mathrm{T}}. \end{cases} \tag{E12}$$

Node p_{38} is generated by rotating node p_2:

$$\mathbf{p}_{38} = \mathbf{R}_3 \mathbf{p}_2. \tag{E13}$$

Node p_{39} is determined by the three-distance intersection operator defined in Equation (E4):

$$\mathbf{p}_{39} = T_s\left(\mathbf{p}_{38}, \mathbf{p}_1, \mathbf{p}_2; L_{39,38}, L_{39,1}, L_{39,2}\right). \tag{E14}$$

After node p_{39} is obtained, node p_{40} is calculated from the parallelogram relation:

$$\mathbf{p}_{40} = \mathbf{p}_{39} + \mathbf{p}_2 - \mathbf{p}_1. \tag{E15}$$

Node p_8 is calculated by prescribing its x-coordinate as t_2. Let

$$\mathbf{u} = \mathbf{p}_8 - \mathbf{p}_{40}, \qquad \mathbf{v} = \mathbf{p}_2 - \mathbf{p}_{40}. \tag{E16}$$

The coordinate and geometric constraints for $\mathbf{u}$ are

$$\begin{cases} u_x = t_2 - x_{40}, \\ \|\mathbf{u}\| = L_{40,8}, \\ \mathbf{u} \cdot \mathbf{v} = 0. \end{cases} \tag{E17}$$

Thus, node p_8 is obtained as

$$\mathbf{p}_8 = \mathbf{p}_{40} + \mathbf{u}. \tag{E18}$$

Node p_{20} is generated by rotational arraying:

$$\mathbf{p}_{20} = \mathbf{R}_1 \mathbf{p}_8. \tag{E19}$$

Using the three-distance intersection operator, the remaining nodes in the reference sector are sequentially calculated as

$$\begin{cases} \mathbf{p}_7 = T_{s_7}\left(\mathbf{p}_8, \mathbf{p}_2, \mathbf{p}_3; L_{7,8}, L_{7,2}, L_{7,3}\right), \\ \mathbf{p}_6 = T_{s_6}\left(\mathbf{p}_7, \mathbf{p}_3, \mathbf{p}_4; L_{6,7}, L_{6,3}, L_{6,4}\right), \\ \mathbf{p}_5 = T_{s_5}\left(\mathbf{p}_6, \mathbf{p}_4, \mathbf{p}_{20}; L_{5,6}, L_{5,4}, L_{5,20}\right), \\ \mathbf{p}_{12} = T_{s_{12}}\left(\mathbf{p}_6, \mathbf{p}_5, \mathbf{p}_{20}; L_{12,6}, L_{12,5}, L_{12,20}\right), \\ \mathbf{p}_{11} = T_{s_{11}}\left(\mathbf{p}_7, \mathbf{p}_6, \mathbf{p}_{12}; L_{11,7}, L_{11,6}, L_{11,12}\right), \\ \mathbf{p}_{10} = T_{s_{10}}\left(\mathbf{p}_8, \mathbf{p}_7, \mathbf{p}_{11}; L_{10,8}, L_{10,7}, L_{10,11}\right), \\ \mathbf{p}_9 = T_{s_9}\left(\mathbf{p}_8, \mathbf{p}_7, \mathbf{p}_{10}; L_{9,8}, L_{9,7}, L_{9,10}\right). \end{cases} \tag{E20}$$

The nodes in the remaining sectors are obtained by

$$\mathbf{p}_{I_k(i)} = \mathbf{R}_k \mathbf{p}_i, \qquad i = 5, 6, \ldots, 12, \quad k = 1, 2, 3. \tag{E21}$$

Using Equations (E9)-(E21), all nodal coordinates of the FO-BW hybrid bidirectional-winding origami structure can be determined

S8. Nodal Coordinate Generation for the BW-FO Hybrid Mode

The BW-FO hybrid mode is constructed by first prescribing the geometric constraints of a bidirectional-winding origami (BW) sector and then coupling it with a Flasher origami (FO) sector. The complete BW-FO hybrid mode contains 40 nodes, which are divided into four identical sectors, with each sector containing 10 primary nodes.

The node-index mapping for the rotational array is defined as

$$J_k(i) = 1 + \mathrm{mod}(i + 10k - 1, 40), \qquad i = 1, 2, \ldots, 10, \quad k = 0, 1, 2, 3. \tag{E22}$$

The corresponding node in the kth sector is obtained as

$$\mathbf{p}_{J_k(i)} = \mathbf{R}_k \mathbf{p}_i, \tag{E23}$$

where $\mathbf{R}_k$ is the rotation matrix defined in Equation (E7).

The geometric constraints of the reference sector are expressed as

$$\|\mathbf{p}_i - \mathbf{p}_j\| = L_{i,j}, \qquad (i,j) \in E_{\mathrm{BWFO},0}, \tag{E24}$$

where $E_{\mathrm{BWFO},0}$ denotes the crease-line set of the reference sector.

The initial nodes are prescribed as

$$\begin{cases} \mathbf{p}_1 = (a/2,\ a/2,\ 0)^{\mathrm{T}}, \\ \mathbf{p}_2 = \left(t_1,\ a/2,\ \sqrt{a^2 - (t_1 - a/2)^2}\right)^{\mathrm{T}}. \end{cases} \tag{E25}$$

Node p_{32} is generated by rotating node p_2:

$$\mathbf{p}_{32} = \mathbf{R}_3 \mathbf{p}_2. \tag{E26}$$

Nodes p_{33} and p_{34} are determined by the three-distance intersection operator:

$$\begin{cases} \mathbf{p}_{33} = T_s\left(\mathbf{p}_{32}, \mathbf{p}_1, \mathbf{p}_2; L_{33,32}, L_{33,1}, L_{33,2}\right), \\ \mathbf{p}_{34} = T_s\left(\mathbf{p}_{32}, \mathbf{p}_{33}, \mathbf{p}_2; L_{34,32}, L_{34,33}, L_{34,2}\right). \end{cases} \tag{E27}$$

Node p_{35} is obtained from the geometric relation

$$\mathbf{p}_{35} = \mathbf{p}_{32} + 2\mathbf{p}_{34} - 2\mathbf{p}_{33}. \tag{E28}$$

Node p_6 is calculated by prescribing its y-coordinate as t_2. Let

$$\mathbf{u} = \mathbf{p}_6 - \mathbf{p}_5, \qquad \mathbf{v} = \mathbf{p}_2 - \mathbf{p}_5. \tag{E29}$$

The coordinate and geometric constraints for $\mathbf{u}$ are

$$\begin{cases} u_y = t_2 - y_5, \\ \|\mathbf{u}\| = L_{5,6}, \\ \mathbf{u} \cdot \mathbf{v} = 0. \end{cases} \tag{E30}$$

Thus, node p_6 is obtained as

$$\mathbf{p}_6 = \mathbf{p}_5 + \mathbf{u}. \tag{E31}$$

Node p_{16} is generated by rotational arraying:

$$\mathbf{p}_{16} = \mathbf{R}_1 \mathbf{p}_6. \tag{E32}$$

Using the three-distance intersection operator, the remaining nodes in the reference sector are sequentially calculated as

$$\begin{cases} \mathbf{p}_{10} = T_{s_{10}}\left(\mathbf{p}_4, \mathbf{p}_{12}, \mathbf{p}_{16}; L_{10,4}, L_{10,12}, L_{10,16}\right), \\ \mathbf{p}_9 = T_{s_9}\left(\mathbf{p}_5, \mathbf{p}_4, \mathbf{p}_{10}; L_{9,5}, L_{9,4}, L_{9,10}\right), \\ \mathbf{p}_8 = T_{s_8}\left(\mathbf{p}_6, \mathbf{p}_5, \mathbf{p}_9; L_{8,6}, L_{8,5}, L_{8,9}\right), \\ \mathbf{p}_7 = T_{s_7}\left(\mathbf{p}_6, \mathbf{p}_5, \mathbf{p}_8; L_{7,6}, L_{7,5}, L_{7,8}\right). \end{cases} \tag{E33}$$

The nodes in the remaining sectors are obtained by

$$\mathbf{p}_{J_k(i)} = \mathbf{R}_k \mathbf{p}_i, \qquad i = 6, 7, \ldots, 10, \quad k = 1, 2, 3. \tag{E34}$$

Through Equations (E22)-(E34), all nodal coordinates of the BW-FO hybrid bidirectional-winding origami structure can be determined

S9. Example illustrating the use of the atan2 function

The two-argument arctangent function, $\mathrm{atan2}(y, x)$, is used to determine a signed angle while preserving the correct quadrant information. Unlike $\mathrm{atan}(y/x)$, which may lose quadrant information, $\mathrm{atan2}(y, x)$ uses the signs of both arguments to determine the correct quadrant of the angle. In this work, it is used to calculate the signed winding angle between two projected position vectors. Let $\overrightarrow{OC}_{\mathrm{start}}$ and $\overrightarrow{OC}_{\mathrm{end}}$ denote the initial and final position vectors from the center point O to the selected node, respectively. The signed winding angle W from $\overrightarrow{OC}_{\mathrm{start}}$ to $\overrightarrow{OC}_{\mathrm{end}}$ is calculated as

$$W = \mathrm{atan2}\left(\left(\overrightarrow{OC}_{\mathrm{start}} \times \overrightarrow{OC}_{\mathrm{end}}\right) \cdot \mathbf{e}_z, \overrightarrow{OC}_{\mathrm{start}} \cdot \overrightarrow{OC}_{\mathrm{end}}\right) \tag{E35}$$

In Equation (E35), $\left(\overrightarrow{OC}_{\mathrm{start}} \times \overrightarrow{OC}_{\mathrm{end}}\right) \cdot \mathbf{e}_z$ is the signed cross-product term projected along the out-of-plane unit vector $\mathbf{e}_z$, which determines the rotation direction. The dot-product term $\overrightarrow{OC}_{\mathrm{start}} \cdot \overrightarrow{OC}_{\mathrm{end}}$ determines the angle magnitude. Therefore, a positive value of W indicates counterclockwise rotation about $\mathbf{e}_z$, whereas a negative value indicates clockwise rotation.

As a simple example, consider

$$\overrightarrow{OC}_{\mathrm{start}} = (1, 0, 0), \qquad \overrightarrow{OC}_{\mathrm{end}} = (0, 1, 0), \qquad \mathbf{e}_z = (0, 0, 1). \tag{E36}$$

The cross-product term is

$$(1, 0, 0) \times (0, 1, 0) \cdot (0, 0, 1) = 1, \tag{E37}$$

and the dot-product term is

$$(1, 0, 0) \cdot (0, 1, 0) = 0. \tag{E38}$$

Thus, the winding angle is obtained as

$$W = \mathrm{atan2}(1, 0) = 90^\circ. \tag{E39}$$

This result means that $\overrightarrow{OC}_{\mathrm{end}}$ is obtained by rotating $\overrightarrow{OC}_{\mathrm{start}}$ counterclockwise by 90° about $\mathbf{e}_z$. In contrast, if

$$\overrightarrow{OC}_{\mathrm{end}} = (0, -1, 0), \tag{E40}$$

then

$$\left(\overrightarrow{OC}_{\mathrm{start}} \times \overrightarrow{OC}_{\mathrm{end}}\right) \cdot \mathbf{e}_z = -1, \qquad \overrightarrow{OC}_{\mathrm{start}} \cdot \overrightarrow{OC}_{\mathrm{end}} = 0. \tag{E41}$$

The corresponding winding angle becomes

$$W = \mathrm{atan2}(-1, 0) = -90^\circ, \tag{E42}$$

which indicates a clockwise rotation about $\mathbf{e}_z$. This example shows that atan2 can distinguish both the magnitude and direction of the rotation, making it suitable for characterizing bidirectional winding behavior.
